# A Person-Centred Approach to Adolescent Emotion Regulation Motives, Strategies, and Perceived Efficacies

**DOI:** 10.1007/s10802-026-01461-y

**Published:** 2026-04-24

**Authors:** Greg Vukets, Paul E. Jose

**Affiliations:** Victoria University of Wellington, Wellington, New Zealand

**Keywords:** Emotion regulation, Adolescents, Mental health, Longitudinal, Process model, Person-centred

## Abstract

The present research utilised the new Process of Emotion Regulation Measure (PERM) to assess emotion regulation motives, selected strategies, and perceived efficacies in a longitudinal community sample of 951 adolescents. We conducted Latent Profile Analyses (LPA) to identify distinct emotion regulation styles and random intercept-latent transition analysis (RI-LTA) to reveal rates of stability and change of profile membership over time. Both LPA and RI-LTA suggested the optimal profile solution was constituted by five emotion regulation profiles: hypo-regulating, hyper-regulating, adaptive, maladaptive, and normative. Each profile evidenced a unique relationship to mental health outcomes, both concurrently and longitudinally. Valenced emotion regulation patterns (evident in the adaptive and maladaptive groups) appeared to manifest the strongest relationships with adolescent mental health. As expected, maladaptive emotion regulation styles were related to poorer wellbeing outcomes, and adaptive emotion regulation styles were related to better wellbeing outcomes. Using a hypo- or hyper-regulating style appeared to influence deviations from the norm for negative outcomes such as anxiety and depression, but they had a minimal influence on positive outcomes such as optimism and resilience. RI-LTA exhibited a general trend toward adaptiveness and showed that unhelpful emotion regulation styles were considerably more transient than helpful emotion regulation styles over a five-month period. Our findings underscore the importance of utilising process-based measures and person-centered analyses when studying adolescent emotion regulation.

**Supplementary Information:**

The online version contains supplementary material available at 10.1007/s10802-026-01461-y.

## A Person-Centred Approach to Adolescent Emotion Regulation Motives, Strategies, and Perceived Efficacies

*Ka* *pū te ruha, ka hao te rangatahi*

*As the old net withers, it is cast aside, and the new net goes fishing*

This Māori whakataukī (proverb) refers to the importance of investing in the next generation, so that they can grow into leaders and continue the work of the community. Despite the widely accepted importance of investing in our rangatahi (youth), adolescent mental wellbeing in New Zealand worsened from 2012 to 2019 (Sutcliffe et al., 2023) and is alarmingly poor on a global scale, recently ranking 36th out of 36 OECD countries with available data (UNICEF, 2025). Moreover, literature suggests that global adolescent mental health may have been worsened or exacerbated by the COVID-19 pandemic, resulting in increased internalising problems (Panchal et al., 2023; Wolf & Schmitz, 2024) and even long-lasting neurological changes (Gotlib et al., 2023).

Emotion regulation (ER) prominently stands out as a key psychological mechanism that is consistently related to, and possibly causative of, both positive and negative mental health outcomes in adolescents (Daniel et al., 2020; Kraft et al., 2023). ER has also importantly been identified as modifiable through different forms of intervention (Eadeh et al., 2021; Moltrecht et al., 2021). It is therefore imperative to delve into the complex relationship between adolescent ER and mental health, with the greater goal of helping our adolescents thrive.

ER is defined by Gross (1998) as attempts to influence which emotions one has, when one has them, and how one experiences or expresses these emotions. Gross’ Extended Process Model (EPM; 2015) conceptualises ER as a three stage process: the *identification* of a goal or motive, the *selection* of a general ER strategy (e.g., choosing cognitive reappraisal or rumination), and the *implementation* of that strategy in a specific and contextual manner. This sequential process model asserts that a goal or motive is the genesis of the ER process, directly constraining strategy selection/use and their accrued outcomes. ER goals can involve seeking to experience a particular emotion (a ‘hedonic goal’) or seeking to experience a particular emotion as a means to a desired end (an ‘instrumental goal’), such as performing a task well or fulfilling a social norm (Tamir, 2016). Hedonic goals can be further divided into ‘pro-hedonic’ goals (seeking to increase positive and decrease negative emotions) and ‘contra-hedonic’ goals (seeking to increase negative and decrease positive emotions).

Research exists on each stage of the EPM’s ER process. At the *identification* stage, contra-hedonic goal-seeking has been linked to increased depression, fear of happiness, and anxiety, as well as decreased gratitude, optimism, and resilience (Bloore et al., 2020) However, this study featured emerging adults – the empirical literature on adolescent ER motives remains scant at this juncture. At the *selection* stage, Gross (2015) suggests ER may ‘fail’ due to numerous potential factors, such as a limited ER strategy repertoire or low ‘ER self-efficacy’ (i.e., one’s belief in their ability to employ a specific ER strategy). These areas have received considerable attention in recent literature: increased ER repertoires and ER self-efficacies have been linked with positive socioemotional functioning, positive affect, and improved ER outcomes (Bassi et al., 2018 Di Gunta et al., 2022; Haag et al., 2024). At the *implementation* stage, increased use of adaptive strategies such as cognitive reappraisal and acceptance has been consistently linked to positive outcomes in adolescents (Aldao et al., 2010). In an opposite direction, increased use of maladaptive ER strategies, such as suppression and avoidance, has been associated with higher rates of psychopathology and problem behaviours (Compas et al., 2017; Schäfer et al., 2017). Existing research has also captured the efficacies of adolescent ER implementations: poor adolescent ER efficacies have been associated with decreased life satisfaction (Henry et al., 2016) and increased risk for a wide range of psychopathologies (McLaughlin et al., 2011).

While this literature is helpful in understanding linkages between ER and mental health, most existing studies have only focused on one stage of the ER process at a time. Gross (1998, 2015) has long emphasised that ER is a process which unfolds over several discrete phases or stages in real time. Research has only recently begun investigating the presumed linkages between ER stages, such as the constraining influences of ER motives on subsequent strategy use (Millgram et al., 2015; Shao et al., 2025). Investigating these ER interrelationships has arguably been hampered by the lack of a comprehensive ER process measure (Mazefsky et al., 2021), as existing ER measures typically operationalise only one stage of the ER process.

To address this need, Vukets and Jose (2026) recently created the Process of Emotion Regulation Measure (PERM). Guided by the EPM’s sequential model, the PERM captures ER goals, strategy selections, and perceived efficacies at the trait-level within the same psychometric instrument. The authors’ initial analyses of PERM subscales evidenced excellent internal consistency, and they obtained good model fit indices for predicted subscale models. Further, latent variable path modelling found convincing evidence for the manifestation of ER across two distinct valenced channels. Within the ‘adaptive’ channel, pro-hedonic motives predicted adaptive strategy use, which, in turn, predicted perceived efficacy of those adaptive strategies. Within the ‘maladaptive’ channel, contra-hedonic motives predicted maladaptive strategy use, which, in turn, also predicted perceived efficacy of those maladaptive strategies (Vukets & Jose, 2026). These variable-centred analyses answered many questions about how EPM stages are related to each other both concurrently and longitudinally, but they lack the ability to inform the field about individual differences in ER styles, or how those individual differences relate to mental health outcomes.

### Person-Centred Research in Adolescent Emotion Regulation

In addition to the need for a comprehensive, process-based ER measure, previous research has also called for increased person-centred work in adolescent ER research (Fombouchet et al., 2023). While understudied, several studies have explored this area to date. Laible et al. (2010) used latent profile analysis (LPA) to identify four distinct adolescent profiles, with each profile varying in levels of self-reported negative emotionality and ER strategy use. Adolescents who were high in negative emotionality, but low in regulation, reported difficulties with anger, aggression, and distress. Nevertheless, these adolescents demonstrated average levels of prosocial behaviours. In contrast, adolescents who were both low in negative emotionality and low in regulation reported the fewest positive social behaviours, and were the least well-adjusted of the four profiles overall.

Turpyn et al. (2015) also used LPA to identify four ER profiles, with each profile describing a way in which adolescents may respond emotionally to conflict with their parents. The researchers found that higher levels of adolescent reactivity and suppression were associated with depression, anxiety, and critical parenting behaviours.

Similarly, Dixon-Gordon et al. (2015) used latent class analysis (LCA) to identify five distinct ‘repertoires’ of ER strategies: ‘low-regulators’ (low use of all strategies), ‘high-regulators’ (high use of all strategies), ‘adaptive regulators’ (higher use of adaptive strategies), ‘worriers/ruminators’, and ‘avoiders’. As indicators for these profiles, the authors assessed three putatively adaptive ER strategies (acceptance, cognitive reappraisal, and problem solving) and four putatively maladaptive strategies (avoidance, suppression, self-criticism, and worry/rumination). The authors found higher levels of psychopathology in the ‘high-regulator’ and ‘worry/ruminate’ classes. While the link between high ER strategy use and psychopathology may be initially surprising, Dixon-Gordon et al. (2015) point out that those individuals with higher levels of psychopathology may simply experience more unwanted emotions, and subsequently require more down-regulation efforts. This study involved undergraduate students rather than secondary school students, but provided provocative person-centred results in a late adolescent sample.

While under-represented, several studies have also explored ER or coping longitudinally through the latent transition analysis (LTA) approach. Amai and Hojo (2022) found that combining help-seeking behaviours with an active coping style was associated with greater school adaptation and affect balance in Japanese adolescents. In a sample of emerging adults, Brewer et al. (2016) determined that the use of cognitive reappraisal and suppression predicted future membership and class transitions of four psychosocial functioning profiles: ‘well-adjusted’, ‘average’, ‘coping with distress’, and ‘maladjusted’. In other words, utilising cognitive reappraisal at Time 1 predicted positive psychosocial adjustment at Time 2. Utilising suppression at Time 1, on the other hand, predicted lower life satisfaction, decreased coping, and increased dysfunctional attitudes at Time 2.

### Developmental Dynamics of Adolescent Emotion Regulation

Adolescence is defined by the World Health Organization (WHO, n.d.) as the phase of life between childhood and adulthood, from ages 10 to 19. This broad age range is characterised by immense biopsychosocial changes, coupled with marked changes in the adolescent’s environment (Blakemore et al., 2010; Casey et al., 2010; Del Piero et al., 2016). It is also characterised by significant developments in ER (Ahmed et al., 2015), and studies have found a general trend toward increased adaptive strategy use (Garnefski & Kraaij, 2006) and strategy effectiveness (Theurel & Gentaz, 2018) throughout adolescence. Interestingly, some studies have also suggested a maladaptive ‘shift’ during middle adolescence (about 13–15 years of age), indicated by the increased use of maladaptive strategies (Cracco et al., 2017; Zimmerman & Iwanski, 2014).

Overall, developmental changes in ER across adolescence remains a relatively underexplored area – particularly the study of hedonic ER goals. It is vital to further our understanding of how ER stages progress across the crucial developmental period of adolescence. Examining how discrete person-centred ER styles manifest over time allows us to progress our understanding of ER as a developmental process, rather than a series of isolated ‘snapshots’. Furthermore, examining how these ER styles differ in mental health outcomes provides a potential framework of individualising ER assessment and intervention, as well as predicting future outcomes (Laursen & Hoff, 2006). Accordingly, the present study employed a random intercept-latent transition analysis (RI-LTA) approach with longitudinal data in order to explicate how these ER styles manifest over time in a person-centred fashion.

### The Present Research

The current study was designed to afford an improved understanding of adolescent ER in three ways. First, we utilised a person-centred analysis of ER goals, strategy selections, and perceived efficacies (as suggested by the EPM and the PERM) to identify novel and distinct profiles of ER in adolescents. In adopting this approach, we expected the profiles to provide insights on the adolescent ER *process*: the linkages among motives, strategies, and perceived efficacies. Second, by examining how these ER profiles predicted concurrent and longitudinal mental health outcomes, we would gain new information about how individual differences in ER style are associated with key psychological outcomes. And third, by analysing the persistence and transition rates of these profiles over time, we would newly appreciate the rates of change and stability in these ER styles during the formative period of adolescence.

We proposed three main hypotheses for this study. First, consistent with Bloore et al.’s (2020) findings, we expected latent profile analysis to yield a three-profile solution: a normative adaptive group, a smaller maladaptive group, and a non-regulating group that fails to strive for outcomes of either valence. Second, we expected MANOVA analyses to show significantly better mental health outcomes in the adaptive group, compared to the maladaptive and non-regulating groups. Finally, we expected RI-LTA to reveal that the adaptive profile would be more stable over time than the maladaptive and non-regulating profiles. We expected this pattern due to previous literature suggesting that adaptive ER improves adolescents’ long-term wellbeing, likely forming a positive feedback loop in which adaptive ER is reinforced and retained (Martínez-Líbano et al., 2025; McLaughlin et al., 2011; Vukets & Jose, 2026).

## Method

### Participants

We collected data at two timepoints in 2024 approximately five months apart, and a longitudinal dataset drawn from 951 participants was obtained. Participants were recruited through convenience sampling from seven schools in Auckland, New Zealand. Students ranged in grade from Year 7 to Year 13, and ages ranged between 10 and 19 years old (M = 14.21, SD = 1.64) at T1. Gender was about evenly distributed (44.1% female, 52.2% male, 3.7% non-binary or did not report), and the sample was composed of 44.5% New Zealand European, 18.3% Asian, 12.2% Māori, 9.0% Pasifika, and 42.0% other ethnicities (allowing for multiethnic self-identification). As a relative measure of socioeconomic status, we used the Equity Index (EQI) of each participant’s school. The EQI is a nation-wide approximation of socio-economic barriers students face to educational achievement. EQI scores fall into seven band ranges, ranging from band 1 – *fewest* barriers, to band 7 – *most* barriers. Our seven schools fell across five of these seven bands, with scores ranging from 398 (“fewest” barriers; band one of seven) to 519 (“many” barriers; band six of seven), with a mean of 443 and median of 430. Our mean participant EQI was 429, falling in the “below average” band range (band three of seven), indicating slightly fewer socioeconomic barriers than the national average of 463.

### Procedure

This research received approval from the Victoria University of Wellington Human Ethics Committee on 20/12/2023 (ID: #31324). We opted for a passive consent procedure to ensure that our data were as representative of New Zealand adolescents as possible by capturing the full range of psychological functioning, rather than a sample skewed toward greater adaptation, typical of adolescent active consent samples (Liu et al., 2017). We viewed our procedure as posing negligible potential risk; in fact, we believe that the procedure was beneficial to teenagers in that it made the ER decision-making process overt and available to conscious evaluation and improvement.

We contacted 15 diverse schools across the Auckland region through email, inviting their participation in our study. After obtaining organisational consent from seven school principals, we worked with senior leadership teams to identify teachers willing to assist with the facilitation of the survey. Students were then informed of the study by their teachers approximately one week in advance of the study’s inception, and provided an information sheet to read and take home to their caregivers. This information sheet was also emailed to caregivers, in the event that the student was absent or did not pass the paper copy to their caregiver/s. On the day of the study, students were again informed of the study, this time by the researcher. Students were then invited to ask any questions, and reminded that they can opt out of the study at any time. Following fair and full informed consent, students were invited to participate through a secure online site. Students took approximately 15–40 minutes to complete the online survey during a typical classroom period under supervision of the teacher and a researcher.

Out of roughly 1000 students approached, only two parents pre-emptively withdrew their child from the study, and no parent concerns were received after data collection. We consider this methodology to be a positive demonstration of the potential benefits and minimal risk of utilising a passive consent procedure in adolescent research.

After a period of approximately five months, the same classes completed the online survey, under supervision of the same researcher. We chose this time period in order to enhance retention of the longitudinal sample by collecting data within a single school year. Following previous literature, we also believed that this would be a sufficient length of time to capture changes in adolescent ER and mental health (Martinsone et al., 2022; McLaughlin et al., 2011).

### Measures

#### Emotion Regulation (PERM)

The Process of Emotion Regulation Measure (PERM) (Vukets & Jose, 2026) is an application of Gross’ Extended Process Model (EPM; 2015) and is divided into three stages: *goal*, *strategy*, and *perceived efficacy*. The 40-item *goal* section asks participants how often they try to experience or avoid experiencing ten positive and ten negative emotions. Responses are given to items such as “In your day-to-day life, how often do you *try* to experience gratitude?” on a five-point Likert scale, ranging from 1 = *Never* to 5 = *Always*. The PERM-*Goal* section is equally divided into two main subscales: pro-hedonic goals and contra-hedonic goals. The pro-hedonic subscale is a mean composite score of how often participants try to experience positive emotions, and try to avoid experiencing negative emotions. The contra-hedonic subscale is a mean composite score of how often participants try to experience negative emotions, and try to avoid experiencing positive emotions.

The 40-item *strategy* section of the PERM asks participants: “What do you typically do when you experience an emotion that you do *not* want to feel?”. Answers to items such as “*Bottle it up*” are given on a five-point Likert scale from 1 = *Never* to 5 = *Always*. The 40-item *efficacy* section asks: “When you use these strategies, do they *help* you in achieving your goal?”, followed by the same 40 items of the strategy section. Answers to items such as “*Try to accept the situation*” are again given on a Likert scale ranging from 1 = *Never* to 5 = *Always*, in addition to an option of 0 = *I never do this.* Both latter PERM sections comprise ten four-item subscales assessing ten distinct ER strategies: distraction, rumination/worry, avoidance, blame, expressive suppression, acceptance, mindfulness, reappraisal, emotional expression, and problem-solving. The former five strategies are putatively maladaptive, and the latter five strategies are putatively adaptive. Collapsing these five strategies therefore provides two main subscales per section: adaptive strategies, maladaptive strategies, adaptive strategy efficacy, and maladaptive strategy efficacy. These main subscales, as well as the pro- and contra-hedonic subscales, have all evidenced excellent internal consistency (αs = 0.91 to 0.96; Vukets & Jose, 2026). Items for all three PERM sections are averaged to create a mean composite score, ranging from 1 to 5.

#### Emotion Dysregulation (DERS)

To measure emotion dysregulation, we administered the 16-item Difficulties in Emotion Regulation Scale (DERS-16; Bjureberg et al., 2016). Using a five-point Likert scale from 1 = *Almost never* to 5 = *Almost always*, participants answer items such as “*I am confused about how I feel*”. Items are summed to create a total score, with higher scores indicating greater emotion regulation difficulties. The DERS-16 has shown excellent internal consistency (α = 0.94).

#### Depression, Anxiety and Stress

The 21-item Depression, Anxiety and Stress Scale - Youth Version (DASS-Y; Szabo & Lovibond, 2022) was administered. Participants indicated how true sentences such as “*My hands felt shaky*” had been for them in the past week on a four-point Likert scale ranging from 0 = *Not true* to 3 = *Very true*. The three subscales have all exhibited good internal consistency: α = 0.89 for Depression, α = 0.84 for Anxiety, and α = 0.84 for Stress. However, previous literature has indicated that anxiety and stress items are more strongly associated with the general distress dimension, rather than their domain-specific dimensions (Osman et al., 2012). We therefore opted to use an “anxiety/stress” subscale and a “depression” subscale, using a sum composite score for each.

#### Subjective Happiness

We administered the four-item Subjective Happiness Scale (SHS; Lyubomirsky & Lepper, 1999) to measure happiness. Respondents chose a point on a seven-point Likert scale, indicating what was most appropriate in describing them. For instance, in response to the item “*In general*,* I consider myself*:”, participants answered from 1 = *Not a very happy person* to 7 = *A very happy person*. The SHS is scored on a mean composite score ranging from 1 to 7, and has shown acceptable to excellent internal consistency (α = 0.79 to 0.94).

#### Optimism

To measure optimism, participants completed the six-item Revised Life Orientation Test (LOT-R; Scheier et al., 1994) by indicating their agreement to items such as “*In uncertain times*,* I usually expect the best*” on a five-point Likert scale ranging from 0 = *Strongly disagree* to 4 = *Strongly agree*. Items are summed to create a composite score. The widely administered LOT-R has shown acceptable internal consistency (α = 0.78).

#### Self-Regulation

Self-regulation was captured through the 31-item Short Self-Regulation Questionnaire (SSRQ; Carey et al., 2004). It has exhibited excellent internal consistency (α = 0.92). Respondents indicate their agreement to items such as “*I have a lot of willpower*” on a five-point Likert scale, ranging from 1 = *Strongly disagree* to 5 = *Strongly agree*. The SSRQ has exhibited excellent internal consistency (α = 0.92). We summed all 31 items to create and utilise a composite score.

#### Gratitude

Participants completed the six-item Gratitude Questionnaire (GQ-6; McCullough et al., 2002), responding to items such as “*I am grateful to a wide variety of people*” on a seven-point Likert scale, ranging from 1 = *Strongly disagree* to 7 = *Strongly agree*. Items are then summed to create a composite score, with higher scores indicating greater gratitude. The GQ-6 has shown good internal consistency (α = 0.82).

#### Resilience

The Revised Child and Youth Resilience Measure (CYRM-R; Jefferies et al., 2019) was administered to measure resilience. On a five-point Likert scale ranging from 1 = *Not at all* to 5 = *A lot*, participants respond to 17 items such as “*I feel that I belong at my school.*” All items were then added to create a composite score. The CYRM-R exhibits good internal consistency (αs = 0.82).

### Data Analyses

#### Treatment of Missingness

We randomly administered half of the items for some measures to reduce respondent fatigue and minimise dropout rates. In this way, random item-level *planned missingness* was enacted in our data. Some degree of *unplanned missingness* in the form of skipped items or completion of one of the two timepoints also occurred. For example, 191 students completed T1 but did not complete T2, and 160 students completed T2 but did not complete T1. MCAR analyses indicated that the missing values were missing completely at random (χ^2^(*df*s = 14,110 to 53,829) = 14,065 to 53,701, *p*s = 0.589 to 0.667), allowing Expectation–Maximization imputation (Lin, 2010) to create a complete dataset.

We obtained 828 responses at T1 and 792 responses at T2. Surveys that did not include answers of at least 50% of the administered items at either of the two time points were removed from the study (Zhang & Yu, 2021). Missing items among responses were then imputed, leaving 791 complete surveys at T1 and 759 complete surveys at T2. Following the merging of T1 and T2 into one dataset, attrition was handled through a second round of imputation, yielding a complete longitudinal dataset of 951 participants. A further rationale for our approach can be found in our supplementary materials (see Table S12 and accompanying interpretation of results).

#### Statistical Methods

Analyses were conducted using SPSS (Version 30.0) and Mplus (Version 8.11) statistical software packages. We ran a series of latent profile analyses (LPAs), using as indicators the main six valenced subscales of the PERM: pro-hedonic motives, contra-hedonic motives, adaptive strategy use, maladaptive strategy use, adaptive strategy efficacy, and maladaptive strategy efficacy. Distraction was removed from the maladaptive strategy use and maladaptive strategy efficacy variables, as initial analyses revealed that distraction loaded equally well with the adaptive and maladaptive channels (i.e., it was not discriminant).

We began the series of LPAs with a two-profile solution, noting Akaike Information Criterion (AIC), Bayesian Information Criterion (BIC), Entropy, and Lo-Mendell-Rubin LRT values, and subsequently increased the number of profiles one by one until we reached an non-optimal solution at six profiles. We used multiple metrics to identify the optimal solution, which was then subjected to a multivariate analysis of variance (MANOVA) to identify profile mean group differences on external variables. Profile was the categorical independent variable, and dependent variables included the following positive and negative mental health outcomes: emotion dysregulation, depression, anxiety and stress, gratitude, self-regulation, happiness, optimism, and resilience. Finally, in order to examine stability and change of distinct profile membership over time, we conducted a random intercept-latent transition analysis (RI-LTA). We chose a RI-LTA over a regular LTA as recent literature has argued for its empirical superiority (Muthén & Asparouhov, 2022).

#### Data Availability

The data that support the findings of this study are openly available in Open Science Framework (OSF) at 10.17605/OSF.IO/RBXTW.

## Results

### Descriptive Statistics

As expected, all three stages of the PERM were significantly associated with mental health outcomes in a valenced manner (see Table 1). Adaptive PERM stages (pro-hedonic motives, adaptive strategy use, adaptive strategy efficacy) were positively related to positive outcomes, and were negatively related to negative outcomes. Maladaptive PERM stages (contra-hedonic motives, maladaptive strategy use, maladaptive strategy efficacy) were negatively related to positive outcomes, and were positively related to negative outcomes. These results were listed in our previous study (Vukets & Jose, 2026) and replicated by T2 data (see Supplementary Tables S1 to S4).

**Table 1:** Descriptive statistics and correlations for time 1 study variables

	Prohed Motives	Contra Motives	Adaptive Strats	Maladap Strats	ASE	MSE	Optimism	DERS	Happy	Self-Reg	Dep	Anxiety/Stress	Gratitude
Prohedonic Motives													
Contrahedonic Motives	− 0.20***												
Adaptive Strategies	0.32***	0.13***											
Maladaptive Strategies	0.08*	0.22***	0.13***										
Adaptive Strategy Efficacy (ASE)	0.30***	0.09**	0.63***	0.14***									
Maladaptive Strategy Efficacy (MSE)	− 0.02	0.24***	0.08*	0.55***	0.26***								
Optimism	0.17***	− 0.06	0.28***	− 0.34***	0.25***	− 0.23***							
Emotion Regulation Difficulties (DERS)	− 0.001	0.19***	0.06	0.53***	0.09**	0.37***	− 0.36***						
Happiness (Happy)	0.22***	− 0.19***	0.16***	− 0.35***	0.14***	− 0.24***	0.49***	− 0.42***					
Self-regulation (Self-reg)	0.26***	− 0.15***	0.36***	− 0.28***	0.30***	− 0.16***	0.49***	− 0.47***	0.39***				
Depression (Dep)	− 0.15***	0.36***	− 0.02	0.47***	− 0.002	0.36***	− 0.40***	0.62***	− 0.57***	− 0.41***			
Anxiety/Stress	− 0.09**	0.33***	0.04	0.49***	0.06	0.33***	− 0.31***	0.69***	− 0.45***	− 0.43***	0.72***		
Gratitude	0.28***	− 0.23***	0.26***	− 0.14***	0.23***	− 0.13***	0.37***	− 0.19***	0.52***	0.41***	− 0.43***	− 0.22***	
Resilience	0.24***	− 0.16***	0.27***	− 0.21***	0.28***	− 0.14***	0.38***	− 0.18***	0.50***	0.44***	− 0.37***	− 0.24***	0.60***
Mean	3.43	1.74	2.78	2.88	2.67	2.50	12.35	42.60	3.09	98.73	4.67	12.99	31.67
Standard Deviation	0.61	0.53	0.55	0.65	0.59	0.73	3.56	12.94	0.89	13.32	4.69	8.67	5.12
Test-retest reliability	0.36***	0.40***	0.39***	0.41***	0.33***	0.39***	0.45***	0.47***	0.37***	0.46***	0.40***	0.48***	0.38***

*p <.05. **p <.01. ***p <.001

### Latent Profile Analyses (LPAs)

Table 2 shows the results of T1 LPA outcomes, beginning with the two-profile solution and iterating to the six-profile solution. We discontinued the series of analyses at six profiles due to several criteria: (1) obtaining a LMR *p-*value over 0.05 for the six-profile solution; (2) obtaining a profile of less than 5% of the sample for the six-profile solution; and (3) AIC, BIC, and entropy values plateauing at five profiles. On these bases, we determined that the five-profile solution was the optimal solution.

**Table 2:** Descriptive statistics from latent profile analyses (LPAs) at time 1

	2 Profiles	3 Profiles	4 Profiles	5 Profiles	6 profiles
AIC	10070.48	9813.25	9601.94	9431.78	9296.05
BIC	10162.77	9939.54	9762.24	9626.10	9524.35
Entropy	0.79	0.77	0.82	0.81	0.84
Lo-Mendell-Rubin adjusted LRT	460.49	265.70	220.71	180.39	146.69
*p*-value	0.001	0.571	0.005	0.024	0.062
N for each profile					
P1	752 (79%)	670 (70%)	178 (19%)	85 (9%)	131 (14%)
P2	199 (21%)	217 (23%)	112 (12%)	145 (15%)	81 (9%)
P3		64 (7%)	616 (65%)	554 (58%)	532 (56%)
P4			45 (5%)	75 (8%)	82 (9%)
P5				92 (10%)	93 (10%)
P6					32 (3%)

*AIC * Akaike Information Criterion, *BIC * Bayesian Information Criterion

**Fig. 1:**
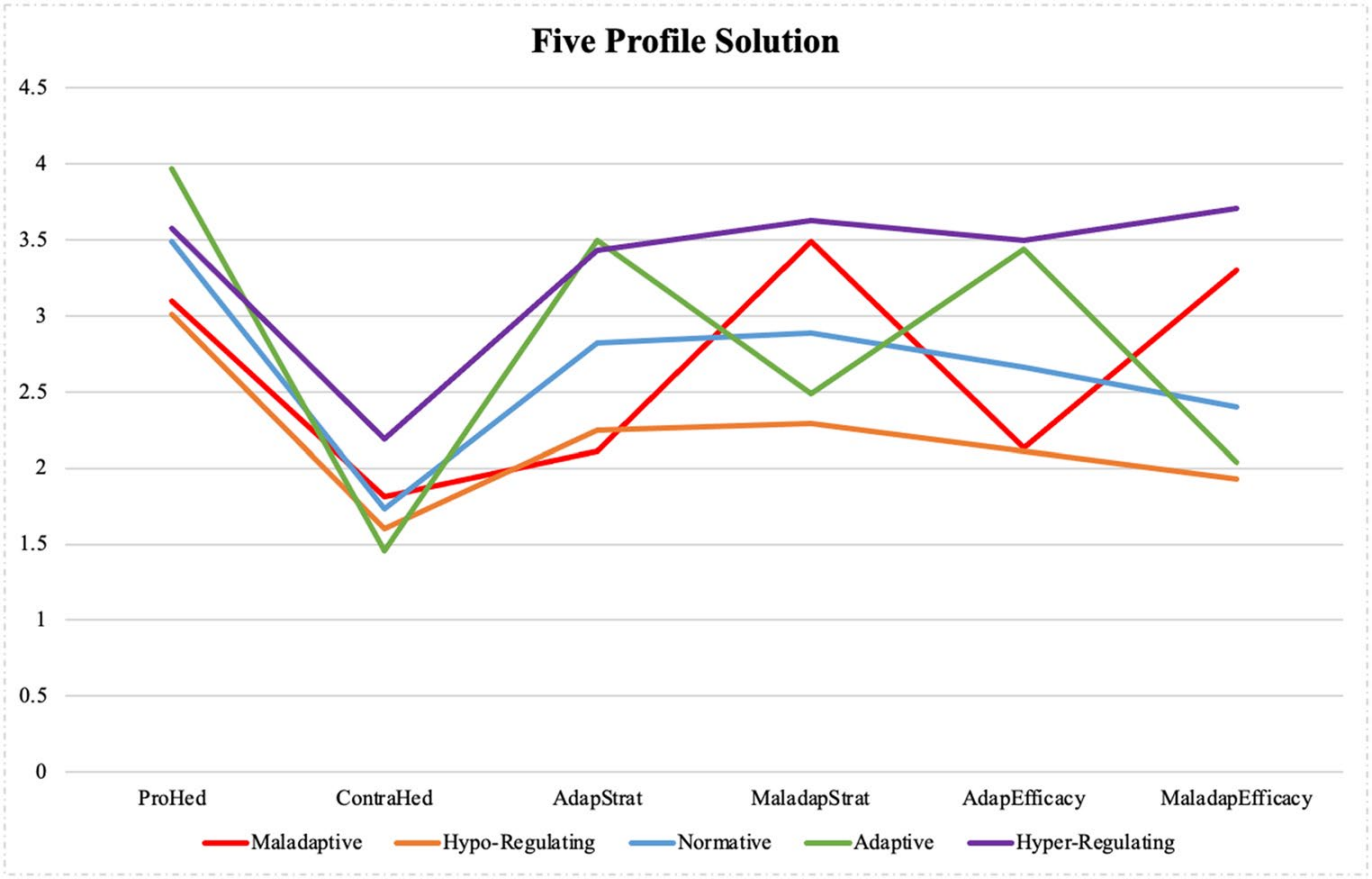
Five profiles graphed across the six valenced emotion regulation indicators

Below is a summary of each of the five profiles (see Fig. 1; Table 3 below for the corresponding graph and variable means):

**Table 3:** Means and standard errors for the PERM facets across profiles

	Maladaptive	Hypo- regulating	Normative	Adaptive	Hyper- regulating	F value	Partial η^2^
Pro-Hedonic Motives	3.10_a_ (0.12)	3.01_a_ (0.11)	3.49_b_ (0.03)	3.97_c_ (0.08)	3.58_b_ (0.07)	58.67	0.20
Contra-Hedonic Motives	1.81_c_ (0.08)	1.60_b_ (0.05)	1.73_c_ (0.03)	1.46_a_ (0.07)	2.19_d_ (0.12)	29.60	0.11
Adaptive Strategies	2.11_a_ (0.07)	2.25_b_ (0.09)	2.82_c_ (0.02)	3.50_d_ (0.07)	3.43_d_ (0.10)	345.43	0.59
Maladaptive Strategies	3.49_d_ (0.10)	2.29_a_ (0.08)	2.89_c_ (0.05)	2.49_b_ (0.16)	3.63_e_ (0.09)	154.78	0.40
Adaptive Strategy Efficacy	2.13_a_ (0.07)	2.11_a_ (0.06)	2.66_b_ (0.03)	3.44_c_ (0.11)	3.50_c_ (0.10)	352.50	0.60
Maladaptive Strategy Efficacy	3.30_c_ (0.09)	1.93_a_ (0.08)	2.40_b_ (0.06)	2.04_a_ (0.11)	3.71_d_ (0.10)	273.34	0.54

Different subscripts reading left to right signify Student-Newman-Keuls (SNK) post-hoc differences at *p* <.01. All *F* values are *p* <.001

**1) Maladaptive group.** This group constituted 9% of the sample. Members of this group evidenced endorsement of higher levels of the maladaptive indicators relative to the adaptive indicators. Maladaptive group members were second lowest in pro-hedonic motive levels (only marginally trailing the hypo-regulating group), and second highest in contra-hedonic motive levels. Adolescents in this group also used maladaptive strategies markedly more often than average, and perceived those strategies to be more helpful in achieving their ER goals than the norm. Furthermore, members of this group used lower levels of adaptive strategies than any other group, and were second lowest in perceiving those strategies as helpful. This profile appears to be similar to the ‘happiness aversion profile’ profile identified by Bloore et al. (2020), evidencing elevated maladaptive indicators in comparison to adaptive indicators.

**2) Hypo-regulating group.** This profile was the second largest group, encompassing 15% of the sample. This group yielded the lowest score in four out of the six indicators, and their scores were consistently lower than the norm across all indicators. Similar to the ‘non-regulating profile’ identified by Bloore et al. (2020), these individuals exhibited a general inability or disinclination to regulate their emotions.

**3) Normative group.** This profile was by far the largest profile within the sample, at 58%. This group yielded means and medians of all six indicators that were very similar to the means and medians for the entire sample. Notably, adolescents in this group used adaptive and maladaptive strategies at a very similar frequency, evidencing a ‘non-valenced’ navigation of ER situations.

**4) Adaptive group.** This group was the smallest of the five profiles, representing 8% of the sample. This group described thriving adolescents: members scored highest in pro-hedonic motives by almost half a point, and simultaneously scored lowest in contra-hedonic motives. They used adaptive strategies the most often, and used maladaptive strategies significantly less often (second only to the hypo-regulating group). Similarly, they perceived adaptive strategies as highly effective, and maladaptive strategies as relatively ineffective. It was notable that the adaptive and maladaptive groups were virtual mirror opposites of each other, particularly for the strategy and perceived efficacy stages.

**5) Hyper-regulating group.** Similar to the adaptive and maladaptive groups, the hyper- and hypo-regulating groups were also near-perfect mirror opposites. At 10% of the sample, this group reported the highest scores for four of the six variables, and all six variable scores were higher than the norm. Hyper-regulators used maladaptive strategies slightly more often than adaptive strategies, and unlike the normative or hypo-regulating groups, they rated these maladaptive strategies as more effective. Also notable was an unusually high contra-hedonic motive score, compared to the other four profiles.

The MANOVA results in Table 3 speak to the robustness of the LPA solution: effect sizes varied from large to extremely large across the six indicators (Fritz et al., 2012). A visual examination of Fig. 1 supports our contention that all five groups evidenced very different and distinctive profiles of ER functioning.

The LPA of T2 data also identified the five-profile solution to be optimal. The means and graphs of T2 LPA profiles conformed very well to the profile characteristics obtained at T1. The BIC value differed very slightly between T1 and T2 solutions (difference = 29.24, or 0.30%). A BIC difference of less than 2.00% is considered to be indicative of LCA invariance (Raftery, 1995), so we interpreted these outputs as evidence for robust replication. Group sizes and means are presented in the Supplemental Materials (see Figure S1 and Table S5).

### Mean Group Differences on Mental Health Outcomes by Profiles

Based on the aforementioned literature showing strong associations between ER with adolescent mental health outcomes, we expected that different profiles (based on PERM indicators) would exhibit significant differences in wellbeing. Specifically, we expected the adaptive group to demonstrate the highest levels of wellbeing, the maladaptive group to demonstrate the lowest levels of wellbeing, and the other groups to demonstrate intermediate levels. Tables 4 and 5 below report individual differences among the five profiles on the eight mental health outcome variables.

**Table 4:** Time 1 means (SDs) and multivariate F-Values for time 1 profile membership correlates

	Maladaptive	Hypo- regulating	Normative	Adaptive	Hyper- regulating	F value	Partial η^2^
Emotion Dysregulation	52.02_c_ (13.34)	35.56_a_ (12.60)	42.07_b_ (11.08)	37.13_a_ (11.43)	52.61_c_ (13.85)	46.90	0.17
Depression	8.62_d_ (6.25)	3.11_b_ (4.03)	4.28_c_ (3.88)	1.96_a_ (2.98)	8.02_d_ (5.65)	45.13	0.16
Anxiety & Stress	18.76_c_ (9.46)	9.61_a_ (8.07)	12.33_b_ (7.42)	9.99_a_ (8.04)	19.45_c_ (10.51)	35.16	0.13
Gratitude	28.15_a_ (5.78)	30.41_b_ (5.64)	32.03_c_ (4.47)	35.59_d_ (4.52)	31.59_bc_ (5.19)	26.45	0.10
Optimism	8.95_a_ (3.81)	12.20_b_ (3.53)	12.59_b_ (3.04)	15.31_c_ (3.61)	11.83_b_ (3.72)	38.85	0.14
Happiness	14.89_a_ (4.45)	18.05_c_ (3.72)	18.04_c_ (3.41)	21.16_d_ (4.08)	16.40_b_ (4.19)	32.62	0.12
Resilience	57.19_a_ (10.11)	63.14_b_ (9.74)	64.29_b_ (8.94)	72.25_c_ (8.68)	64.30_b_ (11.19)	26.08	0.10
Self- regulation	86.44_a_ (14.02)	96.55_b_ (13.48)	99.56_b_ (11.69)	111.31_c_ (12.90)	98.29_b_ (11.56)	42.73	0.15

Different subscripts signify Student-Newman-Keuls (SNK) post hoc test differences at *p* <.01. All *F* values are *p* <.001

**Table 5:**
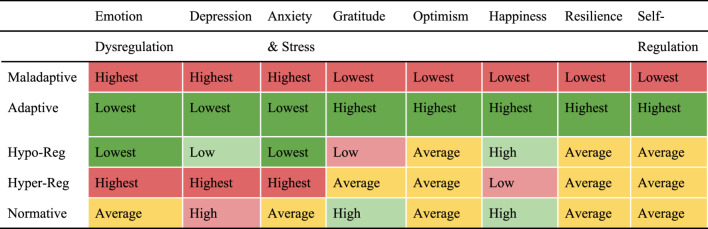
Summaries based on time 1 Student-Newman-Keuls (SNK) post hoc test differences

The colours signify positive and negative outcomes, with the deepest red demonstrating the worst outcomes, yellow signifying average outcomes, and the deepest green representing the best outcomes

Significant differences (medium to large effect sizes) among the five profiles were obtained for all eight outcome variables. As expected, the adaptive group yielded the highest levels of positive wellbeing constructs and the lowest levels of negative wellbeing constructs compared to other groups. In the opposite direction, the maladaptive group expectedly scored lowest on positive constructs and highest on negative constructs, compared to other groups.

The other three groups evidenced distinctive and mixed patterns of positive and negative outcomes intermediate to the two extreme groups. Using a hypo- or hyper- regulating style appeared to influence deviations from the norm for negative outcomes such as anxiety and depression, but these styles had a minimal influence on positive outcomes such as optimism and resilience. Specifically, the hypo-regulating group scored similarly low to the adaptive group on negative constructs, but similar to the normative group (i.e., lower than the adaptive group) on positive constructs. In a similar fashion, the hyper-regulating group scored similarly high to the maladaptive group on negative constructs, but similar to the normative group (i.e., higher than the maladaptive group) on positive constructs. The normative group scored slightly higher than the hyper- and hypo- regulating groups on some indicators, but was generally near the average of the entire sample for all six indicators. Normative group scores fell in between adaptive and maladaptive group scores across all mental health outcome variables.

These findings were replicated by a MANOVA of T2 profiles on T2 mental health outcomes. Moreover, a separate MANOVA of T1 profiles on T2 mental health outcomes revealed that T1 profile membership appeared to have a sustained and persistent influence on mental health outcomes five months later. While the *F* values and effect sizes were lower at T2, all *F* values were again statistically significant (*p* <.001), and post hoc testing revealed similar patterns between profiles to the T1 MANOVA displayed above (see Supplemental Materials for statistical results).

### Mean Group Differences on Demographic Variables by Profiles

As shown by Table 6 below, MANOVA revealed small, significant differences in age and EQI between profiles. The hypo-regulating group yielded the lowest mean age, and the adaptive group yielded the highest mean age. The hyper-regulating group had a significantly higher mean EQI than all other profiles. All these results were replicated at T2.

**Table 6:** Age and Equity Index (EQI) differences between profiles

Timepoint	Variable	Maladaptive	Hypo- regulating	Normative	Adaptive	Hyper- regulating	Sig.	F value	Partial η^2^
Time 1	**Age**	13.92_a_	13.88_a_	14.41_a_	14.45_a_	14.37_a_	0.002**	4.35	0.02
Time 2	**Age**	14.28_b_	13.72_a_	14.34_b_	14.62_b_	14.40_b_	0.007**	3.57	0.02
Time 1	**EQI**	430.82_a_	428.16_a_	428.00_a_	423.53_a_	440.26_b_	0.002**	4.18	0.02
Time 2	**EQI**	425.67_a_	429.88_a_	427.83_a_	433.32_a_	441.26_b_	0.004**	3.83	0.02

Different subscripts reading left to right signify Student-Newman-Keuls (SNK) post-hoc differences at *p* <.01

We also used a chi-square analysis to compare gender frequencies of each ER profile (see Table 7 below). The maladaptive and hyper-regulating groups possessed more female members than male at both timepoints. The adaptive, hypo-regulating, and normative groups all included more male members than female at both timepoints. These results collectively indicate that increased age, increased socioeconomic status and male gender status are all associated with more adaptive ER dispositions.

**Table 7:** Cross-tabulation of gender across profiles

	T1 Male	T1 Female	T2 Male	T2 Female
Maladaptive	32	49	41	47
Hypo-Reg	71	69	56	35
Normative	310	224	342	285
Adaptive	42	32	28	18
Hyper-Reg	41	45	29	34

### Random intercept latent transition analysis (RI-LTA)

**Fig. 2:**
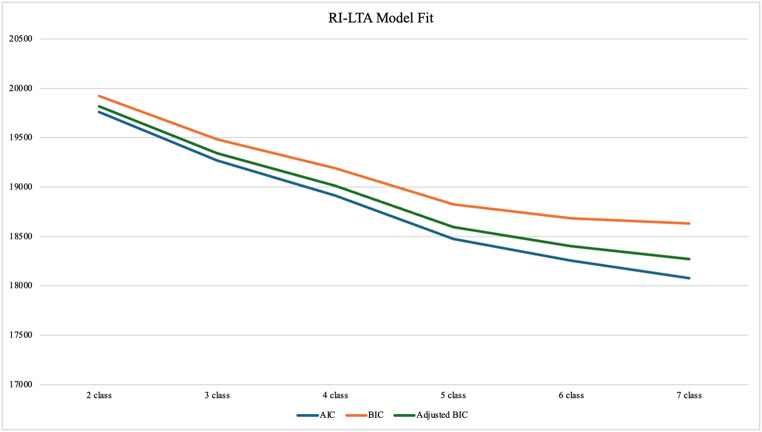
Random intercept-latent transition analysis model fit indices

We ran random intercept latent transition analyses (RI-LTAs) for two to seven classes, recording Akaike Information Criterion (AIC), Bayesian Information Criterion (BIC), Adjusted BIC, and entropy. Graphing these results demonstrated a discernible elbow in these model fit indices at the five-class solution, providing evidence for choosing a five-class solution (see Fig. 2). Notably, plotting the PERM indicator averages of the five RI-LTA profiles yielded a graph virtually identical to the LPA graphs, demonstrating that the nature and patterns of the five profiles were replicated and retained in the longitudinal treatment of the data (see Supplemental Materials Fig. 3). Over time and across all profiles, 58.7% of individuals ‘stayed’ in their profile while 41.3% of individuals ‘moved’ from one profile to another.

 Table8 demonstrates that a general improvement in ER occurred between T1 and T2, with the largest increase being in the adaptive group, and the largest decrease being in the maladaptive group.

**Table 8:** Frequencies of five classes in random-intercept latent transition analysis

Profile	Time 1 Frequency	Time 2 Frequency	Frequency Change
Maladaptive	109 (11.5%)	77 (8.1%)	−32 (−3.4%)
Adaptive	72 (7.6%)	110 (11.6%)	+ 38 (+ 4.0%)
Hypo-Regulating	162 (17.1%)	143 (15.0%)	−19 (−2.1%)
Hyper-Regulating	87 (9.1%)	78 (8.2%)	−9 (−0.9%)
Normative	520 (54.7%)	544 (57.2%)	+ 24 (+ 2.5%)

**Fig. 3:**
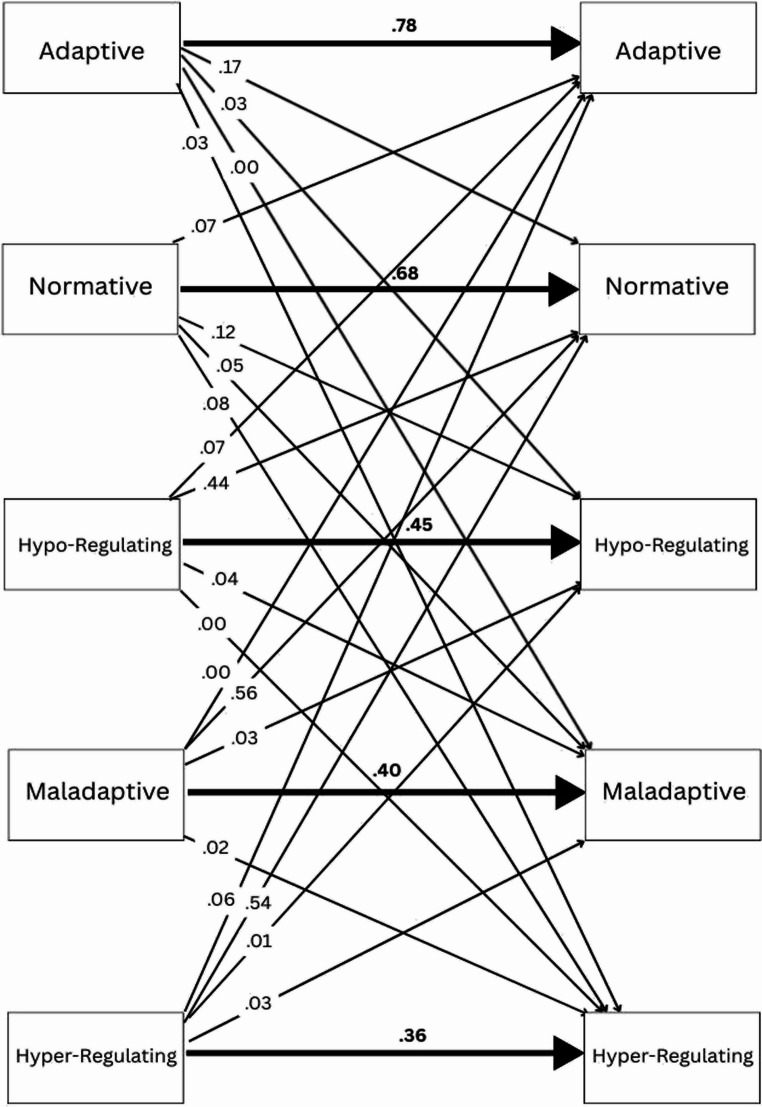
Latent transition probabilities between five classes

As shown by the RI-LTA transition probabilities in Fig. 3 below, the adaptive group was the most stable, with 78% of the T1 group staying in the adaptive group over approximately five months. Among the ‘movers’, most individuals who left the adaptive group moved to the normative group (17%). The normative group was also relatively stable, at 68%. Among those adolescents who left the normative group, the distribution between the other four groups was fairly even, with the hypo-regulating group being the most common destination (12%).

The hypo-regulating, hyper-regulating, and maladaptive groups were all relatively unstable, with less than half of T1 group members staying at T2. Most participants who left these three groups moved to the normative group: in fact, in the maladaptive and hyper-regulating groups, more people left for the normative group than stayed in their initial group. Very little movement was noted among the hypo-regulating, hyper-regulating, and maladaptive groups, suggesting that it was highly uncommon to change between various maladaptive ER approaches over time.

The second most common destination for hypo- and hyper-regulating group movers was the adaptive group (7% and 6% respectively). This result shows that, while uncommon, some adolescents found pathways from irregular, non-valenced ER patterns to adaptive ER patterns. Contrastingly, there appears to be no clear *direct* pathway from maladaptive to adaptive ER (or from adaptive to maladaptive ER).

Further, we examined the role of three demographic covariates on movers and stayers among these profiles: specifically, gender, age, and school Equity Index (a proxy measure for socioeconomic status). A multinomial logistic regression revealed no influence of gender (LRT χ^2^(4) = 5.08, *p* =.28) or Equity Index (LRT χ^2^(4) = 7.96, *p* =.09), but age yielded a significant association (LRT χ^2^(4) = 27.17, *p* <.001). In particular, older adolescents in the T1 maladaptive group were more likely to move to hypo-regulating, hyper-regulating, or normative profiles (LRT Wald values = 3.65 to 7.67, *p*s < 0.001), than younger maladapted adolescents. No other age-related transitions were noted.

## Discussion

The present study reported use of the new Process of Emotion Regulation Measure (Vukets & Jose, 2026) to conduct person-centred analyses of patterns in adolescent ER processes. Our analyses consistently yielded a five-profile solution, consisting of ‘adaptive’, ‘maladaptive’, ‘hypo-regulating’, ‘hyper-regulating’, and ‘normative’ profiles. We tested the concurrent and longitudinal mental health outcomes of each profile over a range of wellbeing measures.

We can identify several key takeaways from these findings. First, large to very large effect sizes in a MANOVA across the five profiles (see Table 3) provided evidence for a very robust person-centred solution. We conclude on this basis that these analyses identified striking and replicable individual differences among a diverse sample of adolescents. In sum, considerable variability seems to exist in how adolescents attempt to manage their emotions.

Second, this variability in ER was shown to be sensibly related to mental health outcomes, both concurrently and longitudinally. Distinctive self-reported ER patterns identified by the PERM (motives, strategies, and efficacies) appear to be strongly predictive of a large range of adolescent mental health indices.

Third, the boundaries between these ER profiles were constrained by two main factors: adolescents ‘striving’ (or not striving) to regulate their emotions, and the consideration of emotion ‘valence’ (operating in adaptive or maladaptive ER channels). The normative, hyper-regulating, and hypo-regulating groups were all relatively non-valenced in their regulation efforts, but differed from each other in the frequency of their motives, strategy use, and perceived efficacies. Adolescents in the hypo-regulating group may have deviated from the norm due to a lack of insight, awareness, or interest in their ER processes. Alternatively, their lower scores may indicate a sense of helplessness, a lack of control, or a lack of knowledge about available ER strategies. Adolescents in the hyper-regulating group, in contrast, may have deviated from the norm due to elevated emotionality (lability), as previously suggested by Dixon-Gordon et al. (2015). Alternatively, following the sequence of the Extended Process Model (EPM; Gross, 2015), their higher ER motive levels may have driven their increased use of strategies, as well as their increased perceived efficacy of those strategies. Further research is required to confirm or reject the aforementioned theoretical rationales for these novel ER profiles.

Fourth, ER profiles were also influenced by several demographic variables. Older age, male gender, and lower EQI (i.e., less barriers to education) were associated with increased membership in normative, hypo-regulating, or adaptive ER groups. These three groups were, in turn, associated with improved mental health outcomes. Contrastingly, female gender and higher EQI were associated with increased membership in the hyper-regulating and maladaptive groups, which evidenced poorer mental health outcomes. These results collectively suggest that lower SES, younger adolescents, and females are more likely to demonstrate unhelpful ER styles, with a subsequent negative effect on their mental health.

Fifth, the extent to which adolescents generally strove to regulate their emotions appeared to have little bearing on mental health outcomes. Despite frequently striving to regulate their emotions, the hyper-regulating group obtained the second-worst outcomes after the maladaptive group, with high depression, anxiety, stress, and emotion dysregulation scores. In contrast, despite striving to regulate their emotions less than any other group, the hypo-regulating group had the second-best outcomes after the adaptive group, with low depression, anxiety, stress, and emotion dysregulation scores. Again, as suggested by Dixon-Gordon et al. (2015), these patterns may reflect underlying emotional lability and severity.

Relative to the amount of striving, the role of desired ER valence evidenced a much stronger role in determining mental health outcomes across all five profiles. The two consistently valenced groups (i.e., the adaptive and maladaptive groups) accrued markedly disparate mental health outcomes: members of the adaptive group reported significantly better mental health outcomes across all measures, while members of the maladaptive group reported significantly worse mental health outcomes across all measures. Further, of the three non-valenced groups, the group which demonstrated more relative maladaptiveness was also the group with the poorest outcomes. Unlike the other two non-valenced groups, the hyper-regulating group yielded higher perceived efficacy scores on the maladaptive channel, compared to the adaptive channel. They also reported a significantly higher contra-hedonic motive score, compared to all other groups. This maladaptive stance likely played a role in the group’s higher depression, anxiety, stress, and emotion dysregulation scores. Thus, valence of ER stages appears to be a strong determining factor behind these results.

Only 17% of the population were consistently preferential in their valenced ER styles, in that they clearly and predominantly operated in either the adaptive or maladaptive channels (i.e., the adaptive and maladaptive groups). Given the clear, strong association between valenced ER styles and mental health outcomes found here, deepening our understanding of the origins of adaptive and maladaptive ER styles is strongly recommended for future directions. Specifically, subsequent research should examine temperamental, experiential, or neurophysiological factors that may contribute to these valenced styles, and replicate or reject our initial findings concerning their transitory or stable natures.

### Change Over Time: Latent Transition Analysis Findings

In addition to these analyses, we also utilised a RI-LTA statistical approach to examine the transition probabilities among the five profiles over five months. Change over this period revealed a small but noticeable general improvement in adolescent ER efforts. Several potential explanations for this trend may be considered. First, normative maturation may have occurred between time points, which generally predicts decreased mood variability during adolescence (Del Piero et al., 2016; Maciejewski et al., 2017). Second, as T2 occurred later in the school year, students may have become more settled and comfortable with their classroom, classmates, and social environments, particularly Year 7 and Year 9 students who would have been attending a new school that year. Third, in completing the survey twice, participants were exposed to customarily undiscussed questions and conversations around ER that may have promoted more self-reflection and conscious decision-making. Completing the survey may have therefore exerted an intervention effect. For example, students may have realised through completing the survey at T1 that they are predominantly using unhelpful strategies such as “bottling it up”, and subsequently endeavoured to improve their ER strategy use between T1 and T2.

RI-LTA also showed that while the adaptive group was relatively small, it was the most stable profile over time. Contrastingly, hyper-regulating and maladaptive profiles were the least stable profiles over time; in fact, more adolescents from these groups moved to the normative group at T2 than stayed in the same group. The maladaptive group correspondingly yielded the largest percentage decrease between T1 and T2, while the adaptive group manifested the largest percentage increase. These provocative findings suggest that maladaptive ER styles appear to be more transient than adaptive ER styles. Once adolescents have established an adaptive ER pattern, they typically maintained this ER style and consequently thrived across an array of wellbeing metrics.

While there was no direct movement to the adaptive group from the maladaptive group, we noted some movement to the adaptive group from the hypo-regulating, hyper-regulating, and normative groups. This pattern suggests that intermediate pathways may exist from non-valenced to adaptive valenced ER patterns. Future research should seek to discern the causal mechanisms that promote adaptive change among these pathways, and the means of increasing their prevalence. Given the stability of adaptive ER, adolescent ER interventions may not necessarily need to focus on maintaining adaptive ER patterns after they are established. Rather, interventions could seek to foster adaptive ER for those adolescents who demonstrate non-valenced or maladaptive ER styles.

### Limitations of the Study

Several shortcomings can be identified in this study. Our convenience sampling method recruited participants from one large urban area in New Zealand. Additionally, mean participant Equity Index (EQI) scores fell slightly below the national mean, indicating slightly lower socioeconomic barriers to education than average. These factors constrain the generalisability of our results. Additionally, we did not elect to analyse the role of ethnicity on the profiles revealed through LPA and RI-LTA.

While heavily influenced by Gross’ sequential EPM (2015), the PERM functions as a trait-level or dispositional measure of ER. Our methodology and the nature of the PERM do not allow for a true evaluation of the sequential EPM, which conceptualises ER efforts over a span of seconds or minutes, rather than months. To directly assess the EPM or PERM’s sequential process, future studies could adopt a short-term, structured, experimental paradigm, such as a video-recorded observational approach. Participants could, for example, respond to an emotion-eliciting task in real time, while relating their associated thoughts and emotions in a structured procedure designed to illuminate how each stage of the EPM informs the ER process.

Further, our analyses centred around the theoretical and dichotomous division of adaptiveness and maladaptiveness. While we found initial evidence for this approach in our previous study (Vukets & Jose, 2026), other research has argued that having a wider repertoire of strategies or using strategies flexibly may be more important, regardless of their valence (Haag et al., 2024; Naragon-Gainey et al., 2017). Finally, as our study is novel in its framework and analytic approach, there is limited explanatory evidence for the rates of movement between profiles identified in our RI-LTA. Future research should test the replicability of these results and explicate the reasons for why some adolescents “stay” in a profile while other adolescents “move” to another profile.

## Conclusions

Despite the widespread acknowledgment of the integral, interconnected role of ER in adolescent mental health, person-centred research on individual differences in adolescent ER is a relatively young and unexplored field. The present research effort is the first known study designed to identify distinct adolescent ER profiles based on valenced indicators of multiple stages of the ER process, rather than an isolated stage (e.g., strategy use). It is also the first known study to utilise a RI-LTA to study rates of change in adolescent ER profiles.

Several potential applications of this research can be mentioned. First, the comprehensive, process-based framework of the PERM enables a newfound understanding of the interrelationships among ER stages, and the relationships of those ER patterns to mental health outcomes. Second, adopting a person-centred approach provides a new means of assessing unique ER patterns within individuals, enabling the identification of idiosyncratic strengths and weaknesses. This assessment approach, in turn, can be utilised to tailor ER interventions to specific individuals or populations, and evaluate the efficacy of those same interventions. We encourage the academic and clinical fields of psychology to consider utilising the PERM and/or person-centred analyses of adolescent ER, given these potential strengths and applications to improving mental health in youth.

### Disclosure of Interest

The authors report there are no competing interests to declare.

## Supplementary Information

Below is the link to the electronic supplementary material.

Supplementary Material 1 (PDF 792 KB)

## Data Availability

The data and materials that support the findings of this study are openly available in Open Science Framework (OSF) at 10.17605/OSF.IO/RBXTW.
