# Supplementary material for: A Person-Centred Approach to Adolescent Emotion Regulation Motives, Strategies, and Perceived Efficacies
Source: Res Child Adolesc Psychopathol. 2026 Apr 24;54(3):66. doi: 10.1007/s10802-026-01461-y (PMC13109273; doi:10.1007/s10802-026-01461-y)
Supplement: Supplementary file 1 — Supplementary Material 1 (PDF 792 KB) [file 10802_2026_1461_MOESM1_ESM.pdf]

# <u>Supplemental Materials</u>

## Table of Contents

Supplementary Description……………………………………………………………………2

Descriptive Statistics…………………………………………………………………..…3

Latent Profile Analysis…………………………………………………………………...7

MANOVAs……………………………………………………………………..………......9

Random Intercept-Latent Transition Analysis………………………………………….…....12

Independent-Samples T Test…………………………………………………………………15

Process of Emotion Regulation Measure (PERM) Full Item List……………………………17

***Supplementary Description***

**Descriptive Statistics.** These include correlation matrices between PERM and mental health outcome variables, both at T1 and T2 (Tables S1 and S2). It also includes means, standard deviations, and Cronbach's alphas for these variables (Tables S3 and S4). Finally, it includes a list of how many participants there were at each age, from 10 to 19 years old.

**Latent Profile Analysis.** This section includes the results for the T2 LPA (T1 results are shown in the main manuscript). This includes means and standard deviations of all six PERM variables for each of the five profiles (Table S5), a graph of those means (Figure S1), and the frequencies of each T2 profile.

**MANOVAs.** Tables S6 to S9 report MANOVAs analysing how T1 and T2 profile membership relates to mental health outcomes – both concurrently and longitudinally.

**Random Intercept-Latent Transition Analysis.** Table S10 provides the main model fit statistics, justifying a five-profile RI-LTA solution (results from this table are graphed in the main manuscript – see Figure 2). Figure S2 depicts the entropy results, providing further evidence for the five-profile solution. Table S11 and Figure S3 report the means of the six main PERM variables (as we did for the LPAs).

**Independent-Samples T Test.** To investigate the effects of attrition, this analysis compared key PERM and mental health variable means between those who *only* completed T1 ("T1 only group") and those who completed both timepoints ("T1 & T2 group"). Table S12 lists all results, and we offer an interpretation for these results. Namely, we discuss how the results confirm the merits of our EM imputation approach for missingness.

**Process of Emotion Regulation Measure (PERM) Full Item List.** As the title suggests, this is a full list of all PERM items used in this study.

## ***Descriptive Statistics***

**Table S1**

*Zero Order Correlations Among Time 2 Variables*

| | Prohed Motives | Contra Motives | Adaptive Strats | Maladap Strats | ASE | MSE | Optimism | DERS | Happy | Self- Reg | Dep | Anxiety/ Stress | Gratitude |
|---|---|---|---|---|---|---|---|---|---|---|---|---|---|
| Prohedonic Motives | —------- | | | | | | | | | | | | |
| Contrahedonic Motives | -.21*** | —------- | | | | | | | | | | | |
| Adaptive Strategies | .33*** | .12*** | —------- | | | | | | | | | | |
| Maladaptive Strategies | .12*** | .23*** | .14*** | —------- | | | | | | | | | |
| Adaptive Strategy Efficacy (ASE) | .21*** | .07* | .58*** | .04 | —------ | | | | | | | | |
| Maladaptive Strategy Efficacy (MSE) | -.06 | .30*** | .08** | .51*** | .29*** | —------- | | | | | | | |
| Optimism | .13*** | -.07* | .31*** | -.31*** | .29*** | -.18*** | —------- | | | | | | |
| Emotion Regulation Difficulties (DERS) | .04 | .24*** | .03 | .58*** | .01 | .36*** | -.32*** | —------- | | | | | |
| Happiness (Happy) | .21*** | -.15*** | .25*** | -.34*** | .24*** | -.20*** | .51*** | -.43*** | —------- | | | | |
| Self-regulation (Self-reg) | .18*** | -.11*** | .39*** | -.27*** | .31*** | -.17*** | .45*** | -.40*** | .37*** | —------- | | | |
| Depression (Dep) | -.10** | .34*** | -.05 | .47*** | -.005 | .39*** | -.40*** | .59*** | -.54*** | -.35*** | —------- | | |
| Anxiety/Stress (Anx) | -.04 | .27*** | -.009 | .47*** | .001 | .33*** | -.25*** | .66*** | -.45*** | -.38*** | .73*** | —------- | |
| Gratitude (Grat) | .29*** | -.26*** | .23*** | -.16*** | .17*** | -.15*** | .37*** | -.25*** | .45*** | .36*** | -.44*** | -.26*** | —------- |
| Resilience (Res) | .30*** | -.19*** | .36*** | -.17*** | .29*** | -.15*** | .40*** | -.22*** | .51*** | .46*** | -.38*** | -.23*** | .59*** |

* $p < .05$, ** $p < .01$, *** $p < .001$

*Note:* Similar to Timepoint 1 (T1), all three stages of the PERM were significantly associated with mental health outcomes in a valenced manner.

# Table S2

*Zero order Correlations Among Time 1 and Time 2 Variables*

| | Prohed Motives1 | Contra Motives1 | Adaptive Strats1 | Maladap Strats1 | ASE1 | MSE1 | Optimism 1 | DERS1 | Happy1 | Self-Reg1 | Dep1 | Anxiety/ Stress1 | Gratitude 1 | Resilience1 |
|---|---|---|---|---|---|---|---|---|---|---|---|---|---|---|
| Prohedonic Motives2 | .36*** | -.11** | .13*** | -.01 | .15*** | -.02 | .12*** | -.06 | .22*** | .17*** | -.15*** | -.11** | .23*** | .18*** |
| Contrahedonic Motives2 | -.15*** | .40*** | .11*** | .11*** | .07* | .18*** | -.02 | .12*** | -.12*** | -.10** | .20*** | .14*** | -.15*** | -.12*** |
| Adaptive Strategies2 | .17*** | .12*** | .39*** | .02 | .34*** | .007 | .17*** | -.01 | .16*** | .22*** | -.06 | -.02 | .14*** | .16*** |
| Maladaptive Strategies2 | -.03 | .10** | -.008 | .41*** | .03 | .31*** | -.20*** | .33*** | -.21*** | -.23*** | .27*** | .31*** | -.12*** | -.10** |
| Adaptive Strategy Efficacy (ASE)2 | .10** | .07* | .25*** | .04 | .33*** | .08* | .12*** | -.01 | .10** | .17*** | .002 | .02 | .07* | .07* |
| Maladaptive Strategy Efficacy (MSE)2 | -.08* | .18*** | .009 | .29*** | .07* | .39*** | -.12*** | .21*** | -.15*** | -.16*** | .20*** | .20*** | -.13*** | -.13*** |
| Optimism2 | .11*** | -.002 | .21*** | -.20*** | .19*** | -.15*** | .45*** | -.17*** | .28*** | .27*** | -.22*** | -.14*** | .18*** | .21*** |
| Emotion Regulation Difficulties (DERS)2 | -.003 | .14*** | -.001 | .31*** | .01 | .25*** | -.19*** | .47*** | -.27*** | -.29*** | .35*** | .40*** | -.12*** | -.12*** |
| Happiness (Happy)2 | .15*** | -.09** | .13*** | -.22*** | .10** | -.17*** | .28*** | -.24*** | .37*** | .23*** | -.31*** | -.25*** | .22*** | .25*** |
| Self-regulation (Self-reg)2 | .12*** | -.06 | .21*** | -.19*** | .16*** | -11*** | .27*** | -.24*** | .21*** | .46*** | -.19*** | -.23*** | .18*** | .21*** |
| Depression (Dep)2 | -.11** | .20*** | .003 | .25*** | .0001 | .22*** | -.21*** | .31*** | -.29*** | -.23*** | .40*** | .34*** | -.23*** | -.19*** |
| Anxiety/Stress (Anx) 2 | -.06 | .16*** | .03 | .29*** | .02 | .23*** | -.16*** | .40*** | -.24*** | -.29*** | .36*** | .48*** | -.15*** | -.13*** |
| Gratitude (Grat)2 | .21*** | -.18*** | .12*** | -.09** | .08** | -.15*** | .20*** | -.14*** | .30*** | .19*** | -.25*** | -.15*** | .38*** | .28*** |
| Resilience (Res)2 | .16*** | -.10** | .14*** | -.13*** | .13*** | -.12*** | .23*** | -.14*** | .27*** | .21*** | -.22*** | -.14*** | .28*** | .37*** |

* *p* <.05, ** *p* <.01, *** *p* <.001

As shown by Table S2, test-retest reliabilities of PERM variables were similar in strength to the other well-established mental health measures. Results demonstrated that all three stages of the PERM were significantly associated with valenced mental health outcomes longitudinally, as well as concurrently.

**Age Ranges of Participants**

10 to 12 years old: 96

13 to 15 years old: 598

16 to 19 years old: 257

*Total: 951*

**Table S3**

*Descriptive statistics for the main PERM subscales.*

| | Time 1 Mean | Time 1 SD | Time 1 α | Time 2 Mean | Time 2 SD | Time 2 α | Test-Retest Reliability |
|---|---|---|---|---|---|---|---|
| Prohedonic Goals | 3.43 | .61 | .92 | 3.47 | .63 | .93 | .36 |
| Contrahedonic Goals | 1.74 | .53 | .94 | 1.72 | .54 | .95 | .40 |
| Adaptive Strategies | 2.78 | .55 | .91 | 2.85 | .58 | .92 | .39 |
| Maladaptive Strategies | 2.88 | .65 | .91 | 2.83 | .64 | .92 | .41 |
| Adaptive Strategy Efficacy | 2.67 | .59 | .93 | 2.75 | .56 | .92 | .33 |
| Maladaptive Strategy Efficacy | 2.50 | .73 | .94 | 2.40 | .70 | .93 | .39 |

**Table S4**

*Descriptive statistics for all mental health measures.*

| | T1 Mean | Time 1 SD | Time 1 α | Time 2 Mean | Time 2 SD | Time 2 α | Test-Retest Reliability |
|---|---|---|---|---|---|---|---|
| Optimism | 12.35 | 3.56 | .64 | 12.71 | 3.57 | .68 | .45 |
| Gratitude | 31.67 | 5.12 | .75 | 32.07 | 4.98 | .78 | .38 |
| Depression | 4.67 | 4.69 | .91 | 4.30 | 4.42 | .91 | .40 |
| Anxiety/Stress | 12.99 | 8.67 | .92 | 12.39 | 8.36 | .92 | .48 |
| Emotion Regulation Difficulties | 42.60 | 12.94 | .94 | 41.72 | 12.53 | .93 | .47 |
| Happiness | 3.09 | .89 | .81 | 3.18 | .89 | .88 | .37 |
| Resilience | 64.11 | 9.87 | .91 | 64.00 | 10.45 | .93 | .37 |
| Self-Regulation | 98.73 | 13.32 | .90 | 100.20 | 13.36 | .90 | .46 |

***Latent Profile Analysis***

**Table S5**

*Means and Standard Errors for PERM Facets across Profiles – Time 2*

| | Maladaptive | Hypo-regulating | Normative | Adaptive | Hyper-regulating | *F* value | Partial $\eta^2$ |
|---|---|---|---|---|---|---|---|
| Pro-Hedonic Motives | $3.16_b$ (.09) | $2.85_a$ (.13) | $3.56_c$ (.03) | $4.03_d$ (.10) | $3.57_c$ (.10) | 63.45 | .21 |
| Contra-Hedonic Motives | $1.83_b$ (.07) | $1.63_a$ (.07) | $1.67_a$ (.03) | $1.49_a$ (.10) | $2.31_c$ (.13) | 31.01 | .12 |
| Adaptive Strategies | $2.25_b$ (.08) | $2.14_a$ (.07) | $2.90_c$ (.03) | $3.75_e$ (.20) | $3.55_d$ (.10) | 294.71 | .56 |
| Maladaptive Strategies | $3.49_d$ (.07) | $2.12_a$ (.08) | $2.80_c$ (.04) | $2.42_b$ (.10) | $3.61_d$ (.09) | 254.09 | .52 |
| Adaptive Strategy Efficacy | $2.26_a$ (.08) | $2.23_a$ (.08) | $2.74_b$ (.03) | $3.62_c$ (.24) | $3.59_c$ (.12) | 159.90 | .40 |
| Maladaptive Strategy Efficacy | $3.13_c$ (.13) | $1.85_a$ (.08) | $2.25_b$ (.04) | $2.21_b$ (.26) | $3.64_d$ (.09) | 203.37 | .46 |

*Note.* Different subscripts reading left to right signify Student-Newman-Keuls (SNK) post-hoc differences at $p < .01$. All *F*-values are $p < .001$.

Probabilities:

1. 646 (67.93%) - Normative
2. 96 (10.10%) - Hypo-regulating
3. 47 (4.94%) - Adaptive
4. 92 (9.67%) - Maladaptive
5. 70 (7.36%) - Hyper-regulating

**Figure S1**

*Five Profiles Graphed Across the Six Valenced Emotion Regulation Indicators – Time 2*

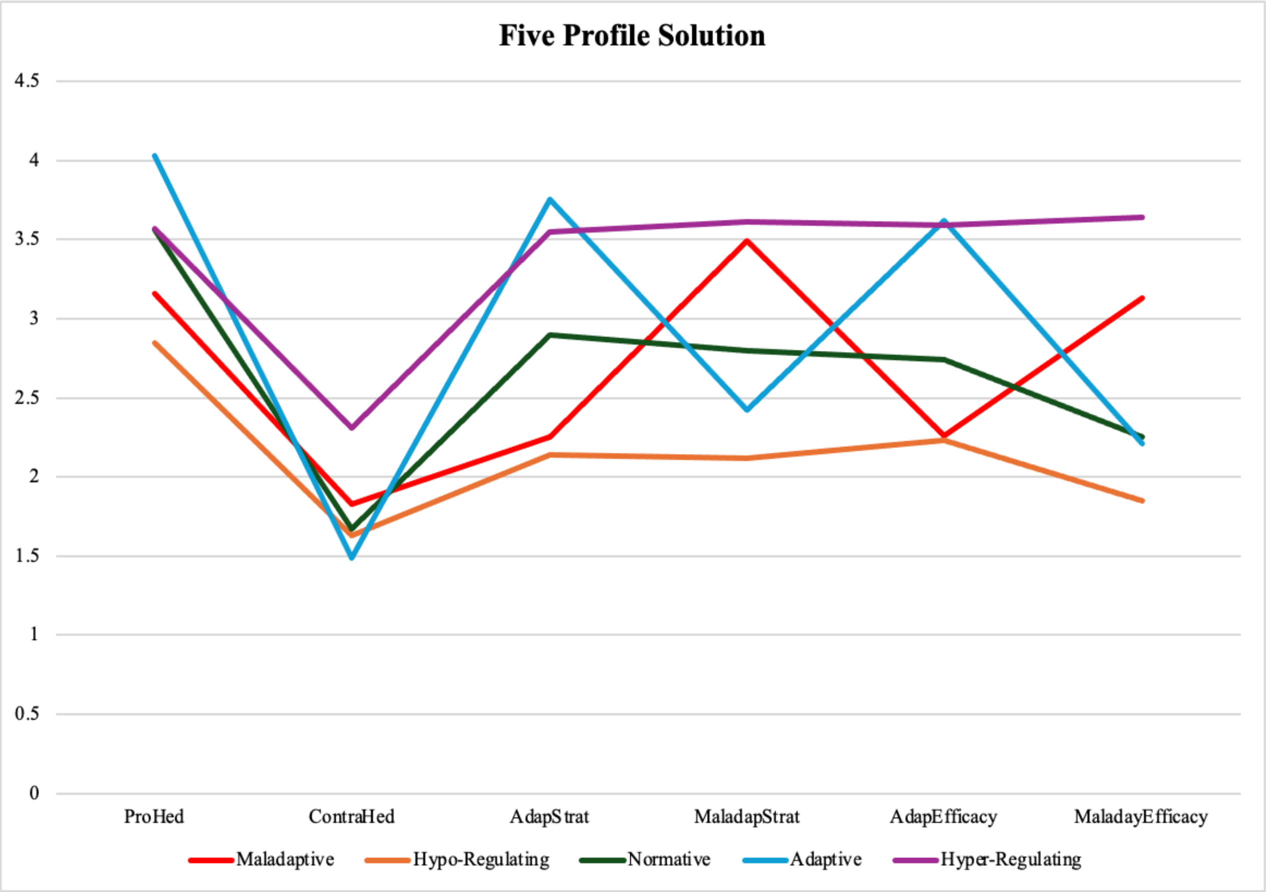


Similar to the T1 LPA, these results demonstrate the distinctiveness of the five ER profiles, with significant differences between groups among all six indicators. T2 LPA yielded the same five distinct profiles: hypo-regulating, hyper-regulating, adaptive, maladaptive, and normative groups. The model fit statistics were similar (Entropy: 0.845; AIC: 9461.04; BIC: 9655.34). However, the probabilities were somewhat different, with smaller adaptive and hyper-regulating groups, and a larger normative group.

***MANOVAs***

**Table S6**

*Time 2 Means (SDs) and Multivariate F Values for Time 1 Profile Membership Correlates*

| | Maladaptive | Hypo-regulating | Normative | Adaptive | Hyper-regulating | *F* value | Partial $\eta^2$ |
|---|---|---|---|---|---|---|---|
| Emotion Dysregulation | $47.25_c$ (12.89) | $37.43_a$ (10.96) | $41.72_b$ (12.17) | $38.24_a$ (12.22) | $46.26_c$ (13.63) | 13.53 | .05 |
| Depression | $6.18_b$ (4.66) | $3.12_a$ (3.40) | $4.18_a$ (4.42) | $2.94_a$ (3.23) | $6.25_b$ (5.21) | 13.43 | .05 |
| Anxiety & Stress | $15.57_b$ (8.77) | $9.81_a$ (6.43) | $12.34_a$ (8.44) | $10.68_a$ (7.45) | $15.15_b$ (9.13) | 10.51 | .04 |
| Gratitude | $30.44_a$ (4.90) | $31.79_{ab}$ (4.92) | $32.21_b$ (5.05) | $34.72_c$ (4.10) | $30.99_{ab}$ (4.52) | 9.20 | .04 |
| Optimism | $10.15_a$ (3.38) | $12.67_b$ (3.24) | $12.88_b$ (3.53) | $14.53_c$ (3.47) | $12.65_b$ (3.39) | 17.25 | .07 |
| Happiness | $16.22_a$ (3.39) | $18.30_b$ (3.30) | $18.38_b$ (4.28) | $20.83_c$ (4.32) | $17.43_b$ (3.82) | 14.19 | .06 |
| Resilience | $60.36_a$ (9.53) | $64.22_b$ (8.51) | $63.69_b$ (10.85) | $70.21_c$ (9.32) | $63.80_b$ (10.49) | 9.70 | .04 |
| Self-regulation | $94.15_a$ (11.56) | $98.94_b$ (12.45) | $100.40_b$ (13.51) | $109.04_c$ (13.88) | $99.39_b$ (11.26) | 13.70 | .06 |

*Note.* * $p$ <.05, ** $p$ <.01, *** $p$ <.001. All $F$ values are $p < .001$.

**Table S7**

*Descriptors from Pair-Wise Comparison Analysis – Time 1 Profiles, Time 2 Outcomes*

| | Emotion Dysregulation | Depression | Anxiety & Stress | Gratitude | Optimism | Happiness | Resilience | Self-Regulation |
|---|---|---|---|---|---|---|---|---|
| Maladaptive | Highest | High | High | Lowest | Lowest | Lowest | Lowest | Lowest |
| Adaptive | Lowest | Low | Low | Highest | Highest | Highest | Highest | Highest |
| Hypo-Reg | Lowest | Low | Low | Average | Average | Average | Average | Average |
| Hyper-Reg | Highest | High | High | Low | Average | Average | Average | Average |
| Normative | Average | Low | Low | Average | Average | Average | Average | Average |

Tables S4 and S5 above show that T1 ER profile membership predicted significant differences in T2 mental health outcomes. Maladaptive and hyper-regulating groups yielded the highest scores on negative outcome measures, while the adaptive and hypo-regulating groups produced the lowest scores on these measures. The maladaptive group yielded the lowest scores on positive outcome measures, while the adaptive group exhibited the highest scores on these measures. The three non-valenced ER groups fell in the average range for positive mental health outcomes.

**Table S8**

*Time 2 Means (SDs) and Multivariate F-Values for Time 2 Profile Membership Correlates*

| | Maladaptive | Hypo-regulating | Normative | Adaptive | Hyper-regulating | *F* value | Partial $\eta^2$ |
|---|---|---|---|---|---|---|---|
| Emotion Dysregulation | $51.87_c$ (14.10) | $34.33_a$ (11.81) | $40.83_b$ (10.50) | $34.97_a$ (13.00) | $51.33_c$ (14.79) | 44.96 | .16 |
| Depression | $8.32_c$ (5.60) | $2.56_{ab}$ (3.64) | $3.73_b$ (3.46) | $1.88_a$ (3.17) | $8.31_c$ (6.42) | 52.88 | .18 |
| Anxiety & Stress | $18.99_c$ (10.48) | $8.25_a$ (6.93) | $11.71_b$ (7.01) | $8.73_a$ (8.05) | $18.21_c$ (10.88) | 36.92 | .14 |
| Gratitude | $28.77_a$ (5.55) | $30.58_b$ (6.03) | $32.53_c$ (4.38) | $35.54_d$ (4.75) | $31.85_{bc}$ (5.40) | 20.99 | .08 |
| Optimism | $9.21_a$ (3.96) | $12.35_b$ (2.94) | $13.07_b$ (3.19) | $15.75_c$ (3.55) | $12.52_b$ (3.95) | 37.54 | .14 |
| Happiness | $14.45_a$ (4.30) | $17.90_b$ (4.32) | $18.67_b$ (3.53) | $21.83_c$ (4.22) | $17.82_b$ (5.28) | 34.33 | .13 |
| Resilience | $55.40_a$ (9.38) | $60.24_b$ (13.42) | $64.81_c$ (8.80) | $74.45_d$ (7.89) | $65.93_c$ (13.04) | 36.85 | .14 |
| Self-regulation | $89.78_a$ (14.90) | $97.05_b$ (12.19) | $100.90_c$ (11.79) | $114.52_d$ (15.15) | $102.24_c$ (14.18) | 33.77 | .13 |

*Note*. Different subscripts signify Student-Newman-Keuls (SNK) post hoc test differences at $p < .01$. All $F$ values are $p < .001$.

**Table S9**

*Time 2 Descriptors from Student-Newman-Keuls (SNK) Post Hoc Test Differences*

| | Emotion Dysregulation | Depression | Anxiety & Stress | Gratitude | Optimism | Happiness | Resilience | Self-Regulation |
|---|---|---|---|---|---|---|---|---|
| Maladaptive | Highest | Highest | Highest | Lowest | Lowest | Lowest | Lowest | Lowest |
| Adaptive | Lowest | Lowest | Lowest | Highest | Highest | Highest | Highest | Highest |
| Hypo-Reg | Lowest | Lowest | Lowest | Low | Average | Average | Low | Low |
| Hyper-Reg | Highest | Highest | Highest | Average | Average | Average | High | High |
| Normative | Average | Average | Average | High | Average | Average | High | High |

*Note.* The colours signify positive and negative outcomes, with the deepest red demonstrating the worst outcomes, yellow signifying average outcomes, and the deepest green representing the best outcomes.

Tables S6 and S7 replicate the T1 analyses provided in the main body of the manuscript. Again, the maladaptive and hyper-regulating groups produced the highest scores on negative outcome measures, while the adaptive and hypo-regulating groups reported the lowest scores on these measures. The maladaptive group yielded the lowest scores on positive outcome measures, while the adaptive group exhibited the highest scores on these measures. Unlike the T1 analyses, the hypo-regulating profile appeared to manifest the worst positive mental health outcome scores of the three non-valenced groups (i.e., compared to the hyper-regulating and normative groups).

***Random Intercept-Latent Transition Analysis***

**Table S10**

*Model Fit Indices for Latent Transition Analyses*

| | 2 class | 3 class | 4 class | 5 class | 6 class | 7 class |
|---|---|---|---|---|---|---|
| AIC | 19762.11 | 19270.66 | 18915.45 | 18474.22 | 18253.18 | 18078.67 |
| BIC | 19922.41 | 19484.39 | 19192.33 | 18823.96 | 18685.50 | 18632.43 |
| Adjusted BIC | 19817.60 | 19344.65 | 19011.30 | 18595.29 | 18402.84 | 18270.37 |
| Entropy | .89 | .82 | .78 | .83 | .84 | .84 |

**Figure S2**

*Entropy Scores from RI-LTA*

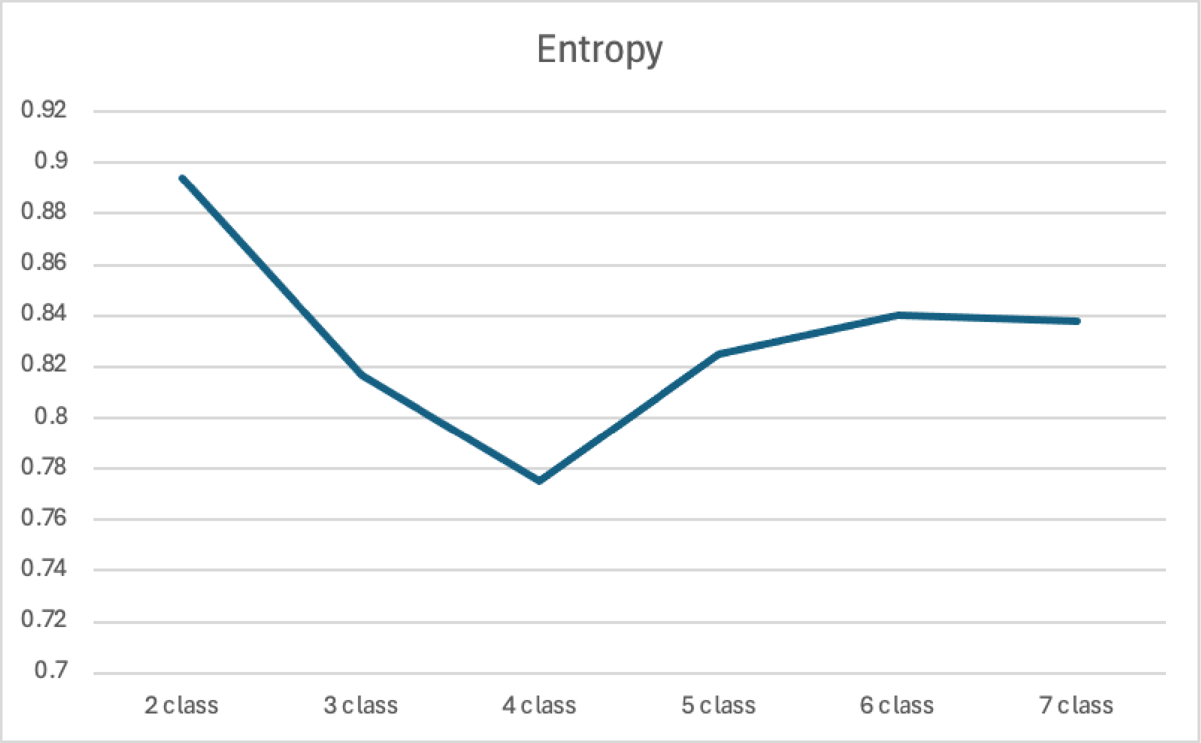


*Note.* The figure above shows a kink and subsequent plateau in entropy at the five-class solution, informing a good model fit for the five-class solution in our RI-LTA. AIC, BIC and adjusted BIC also yielded a kink at five classes – this graph was included in the main text of the manuscript.

**Table S11**

*Means for PERM Facets Across Profiles – Time 1*

| | Hypo-Regulating | Maladaptive | Adaptive | Normative | Hyper-Regulating |
|---|---|---|---|---|---|
| Pro-hedonic Motives | 3.02 | 3.14 | 3.91 | 3.52 | 3.60 |
| Contra-hedonic Motives | 1.59 | 1.78 | 1.41 | 1.73 | 2.26 |
| Adaptive Strategies | 2.31 | 2.20 | 3.46 | 2.85 | 3.45 |
| Maladaptive Strategies | 2.18 | 3.52 | 2.34 | 2.91 | 3.61 |
| Adaptive Efficacy | 2.21 | 2.19 | 3.35 | 2.70 | 3.56 |
| Maladaptive Efficacy | 1.84 | 3.18 | 1.98 | 2.38 | 3.66 |

**Figure S3**

*RI-LTA Profiles Graphed Across the Six Valenced Emotion Regulation Variables*

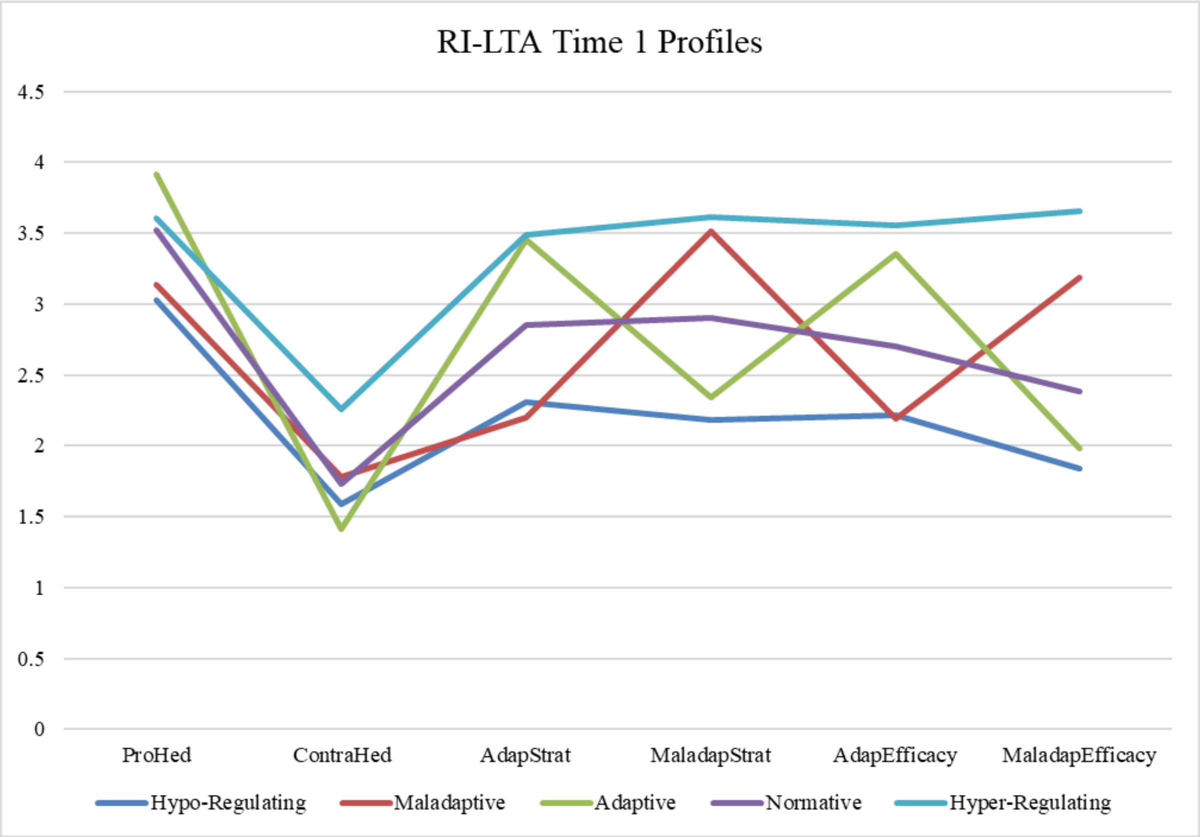

As shown by Table S9 and Figure S3 above, the five-profile RI-LTA solution yielded virtually identical ER profiles to both LPAs (T1 and T2).

***Independent-Samples T Test***

To investigate the effects of attrition, we compared key variable means between those who completed both timepoints and those who *only* completed T1. All statistically significant group differences are shown by Table S10 below.

**Table S12**

*Mean Differences Between Groups by Timepoint Participation*

| Variable | Group Means | Two-Sided *p* | Cohen's *d* |
|---|---|---|---|
| Age | 14.37<br>**14.20** | .21 | .11 |
| Equity Index | 435.71<br>**426.34** | <.001*** | .31 |
| Gender | 1.55<br>**1.43** | .003** | .25 |
| Pro-Hedonic Motives | 3.31<br>**3.45** | .02* | -.20 |
| Contra-Hedonic Motives | 1.88<br>**1.69** | <.001*** | .33 |
| Adaptive Strategies | 2.75<br>**2.78** | .51 | -.06 |
| Maladaptive Strategies | 3.04<br>**2.96** | .16 | .12 |
| Adap Strategy Efficacy | 2.67<br>**2.67** | .99 | -.001 |
| Maladap Strategy Efficacy | 2.69<br>**2.61** | .22 | .11 |
| Optimism | 11.81<br>**12.49** | .05* | -.18 |
| Emotion dysregulation (DERS) | 43.37<br>**42.37** | .40 | .07 |
| Happiness | 17.64<br>**17.91** | .45 | -.06 |
| Self-regulation | 96.30<br>**99.42** | .01** | -.21 |
| Depression, Anxiety & Stress | 19.81<br>**16.97** | .02* | .21 |
| Gratitude | 30.89<br>**31.88** | .04* | -.18 |
| Resilience | 63.06<br>**64.44** | .14 | -.13 |

*Note*. T1 only group means are in normal text, while T1 & T2 group means are in **bold text**.

Notable demographic differences include increased EQI and female membership for the T1 only group. Additionally, chi-square analysis showed that the T1 only group had a markedly greater proportion of Māori (16.1%) and Pasifika (14.5%), compared to the T1 & T2 group (8.4% Māori, 6.7% Pasifika). No significant differences were found for age.

The T1 only group reported decreased pro-hedonic motives and increased contra-hedonic motives, compared to the T1 & T2 group. Relatedly, they also reported decreased optimism, self-regulation, and gratitude, and increase depression, anxiety and stress. No significant differences were found for PERM strategy use, PERM strategy efficacy, emotion dysregulation, happiness, or resilience.

These results provide a further justification for the EM-imputation approach, as allowing listwise deletion would exclude T1 only participants and subsequently introduce significant bias into our data.

***Process of Emotion Regulation Measure (PERM) Full Item List***

*PERM-Goal*

Q1: “In your day-to-day life, how often do you TRY to experience…?”

| | Never (1) | Occasionally (2) | About half the time (3) | Most of the time (4) | All of the time (5) |
|---|---|---|---|---|---|
| Happiness (1) | ○ | ○ | ○ | ○ | ○ |
| Joy (2) | ○ | ○ | ○ | ○ | ○ |
| Pride (3) | ○ | ○ | ○ | ○ | ○ |
| Gratitude (4) | ○ | ○ | ○ | ○ | ○ |
| Love (5) | ○ | ○ | ○ | ○ | ○ |
| Peacefulness (6) | ○ | ○ | ○ | ○ | ○ |
| Compassion (7) | ○ | ○ | ○ | ○ | ○ |
| Hope (8) | ○ | ○ | ○ | ○ | ○ |
| Balance (9) | ○ | ○ | ○ | ○ | ○ |
| Enthusiasm (10) | ○ | ○ | ○ | ○ | ○ |
| Frustration (11) | ○ | ○ | ○ | ○ | ○ |
| Sadness (12) | ○ | ○ | ○ | ○ | ○ |
| Anger (13) | ○ | ○ | ○ | ○ | ○ |
| Shame (14) | ○ | ○ | ○ | ○ | ○ |
| Disgust (15) | ○ | ○ | ○ | ○ | ○ |
| Fear (16) | ○ | ○ | ○ | ○ | ○ |
| Regret (17) | ○ | ○ | ○ | ○ | ○ |

| | | | | | |
|---|---|---|---|---|---|
| Distress (18) | ○ | ○ | ○ | ○ | ○ |
| Anxiety (19) | ○ | ○ | ○ | ○ | ○ |
| Loneliness (20) | ○ | ○ | ○ | ○ | ○ |

Q2: "In your day-to-day life, how often do you try to AVOID experiencing…?"

| | Never (1) | Occasionally (2) | About half the time (3) | Most of the time (4) | All of the time (5) |
|---|---|---|---|---|---|
| Happiness (1) | ○ | ○ | ○ | ○ | ○ |
| Joy (2) | ○ | ○ | ○ | ○ | ○ |
| Pride (3) | ○ | ○ | ○ | ○ | ○ |
| Gratitude (4) | ○ | ○ | ○ | ○ | ○ |
| Love (5) | ○ | ○ | ○ | ○ | ○ |
| Peacefulness (6) | ○ | ○ | ○ | ○ | ○ |
| Compassion (7) | ○ | ○ | ○ | ○ | ○ |
| Hope (8) | ○ | ○ | ○ | ○ | ○ |
| Balance (9) | ○ | ○ | ○ | ○ | ○ |
| Enthusiasm (10) | ○ | ○ | ○ | ○ | ○ |
| Frustration (11) | ○ | ○ | ○ | ○ | ○ |
| Sadness (12) | ○ | ○ | ○ | ○ | ○ |
| Anger (13) | ○ | ○ | ○ | ○ | ○ |
| Shame (14) | ○ | ○ | ○ | ○ | ○ |

| | | | | | |
|---|---|---|---|---|---|
| Disgust (15) | ○ | ○ | ○ | ○ | ○ |
| Fear (16) | ○ | ○ | ○ | ○ | ○ |
| Regret (17) | ○ | ○ | ○ | ○ | ○ |
| Distress (18) | ○ | ○ | ○ | ○ | ○ |
| Anxiety (19) | ○ | ○ | ○ | ○ | ○ |
| Loneliness (20) | ○ | ○ | ○ | ○ | ○ |

*PERM-Strategy*

Q3: People often face situations in life where they experience emotions that they do <u>not</u> want to feel. What do you typically do when you experience an emotion that you do <u>not</u> want to feel?

| | Never (1) | Occasionally (2) | About half the time (3) | Most of the time (4) | Always (5) |
|---|---|---|---|---|---|
| Do something else to distract myself (1) | ○ | ○ | ○ | ○ | ○ |
| Try to change what I am thinking about (2) | ○ | ○ | ○ | ○ | ○ |
| Accept the way things are (3) | ○ | ○ | ○ | ○ | ○ |
| Go over the emotion again and again in my mind (4) | ○ | ○ | ○ | ○ | ○ |
| Bottle it up (5) | ○ | ○ | ○ | ○ | ○ |
| Blame myself for the way I am feeling (6) | ○ | ○ | ○ | ○ | ○ |
| Engage in something else to keep busy (7) | ○ | ○ | ○ | ○ | ○ |
| Take note of how my body was feeling (8) | ○ | ○ | ○ | ○ | ○ |
| Ignore my emotions (9) | ○ | ○ | ○ | ○ | ○ |
| Look for comfort or support from someone (10) | ○ | ○ | ○ | ○ | ○ |
| Look for a positive side (11) | ○ | ○ | ○ | ○ | ○ |
| Dwell upon my feelings (12) | ○ | ○ | ○ | ○ | ○ |
| Criticise myself (13) | ○ | ○ | ○ | ○ | ○ |
| Try to control my feelings, calm down, and relax (14) | ○ | ○ | ○ | ○ | ○ |
| I don't do anything (15) | ○ | ○ | ○ | ○ | ○ |
| Look for solutions (16) | ○ | ○ | ○ | ○ | ○ |
| Try to accept the situation (17) | ○ | ○ | ○ | ○ | ○ |
| Think of things I can do about it (18) | ○ | ○ | ○ | ○ | ○ |
| Make sure no one can tell what I am feeling (19) | ○ | ○ | ○ | ○ | ○ |
| Pretend that there is no problem (20) | ○ | ○ | ○ | ○ | ○ |

| | | | | | |
|---|---|---|---|---|---|
| Try to express my feelings (21) | ○ | ○ | ○ | ○ | ○ |
| Tell myself that it is all my fault (22) | ○ | ○ | ○ | ○ | ○ |
| Try to think about how I can change my situation (23) | ○ | ○ | ○ | ○ | ○ |
| Worry about the way I was feeling (24) | ○ | ○ | ○ | ○ | ○ |
| Hide my emotions (25) | ○ | ○ | ○ | ○ | ○ |
| Sit with my emotions and let them be (26) | ○ | ○ | ○ | ○ | ○ |
| Think about other ways of seeing the situation I am in (27) | ○ | ○ | ○ | ○ | ○ |
| Focus on slowing my heart rate and breathing (28) | ○ | ○ | ○ | ○ | ○ |
| Distract myself with another task e.g. exercise, TV, or music (29) | ○ | ○ | ○ | ○ | ○ |
| Try to fix it (30) | ○ | ○ | ○ | ○ | ○ |
| Tell somebody how I am feeling (31) | ○ | ○ | ○ | ○ | ○ |
| Act as if nothing is going on (32) | ○ | ○ | ○ | ○ | ○ |
| Do something else to think about something new (33) | ○ | ○ | ○ | ○ | ○ |
| Accept my emotions (34) | ○ | ○ | ○ | ○ | ○ |
| Close myself off to others (35) | ○ | ○ | ○ | ○ | ○ |
| Think about a happier time in my life (36) | ○ | ○ | ○ | ○ | ○ |
| Take some deep breaths (37) | ○ | ○ | ○ | ○ | ○ |
| Worry about what I might do (38) | ○ | ○ | ○ | ○ | ○ |
| Show my feelings through words or actions (39) | ○ | ○ | ○ | ○ | ○ |
| Think about the mistakes I have made (40) | ○ | ○ | ○ | ○ | ○ |

*PERM-Efficacy*

Q4: When you use these strategies, do they <u>help</u> you in achieving your goal?

| | Never (1) | Occasionally (2) | About half the time (3) | Most of the time (4) | Always (5) |
|---|---|---|---|---|---|
| Do something else to distract myself (1) | ○ | ○ | ○ | ○ | ○ |
| Try to change what I am thinking about (2) | ○ | ○ | ○ | ○ | ○ |
| Accept the way things are (3) | ○ | ○ | ○ | ○ | ○ |
| Go over the emotion again and again in my mind (4) | ○ | ○ | ○ | ○ | ○ |
| Bottle it up (5) | ○ | ○ | ○ | ○ | ○ |
| Blame myself for the way I am feeling (6) | ○ | ○ | ○ | ○ | ○ |
| Engage in something else to keep busy (7) | ○ | ○ | ○ | ○ | ○ |
| Take note of how my body was feeling (8) | ○ | ○ | ○ | ○ | ○ |
| Ignore my emotions (9) | ○ | ○ | ○ | ○ | ○ |
| Look for comfort or support from someone (10) | ○ | ○ | ○ | ○ | ○ |
| Look for a positive side (11) | ○ | ○ | ○ | ○ | ○ |
| Dwell upon my feelings (12) | ○ | ○ | ○ | ○ | ○ |
| Criticise myself (13) | ○ | ○ | ○ | ○ | ○ |
| Try to control my feelings, calm down, and relax (14) | ○ | ○ | ○ | ○ | ○ |
| I don't do anything (15) | ○ | ○ | ○ | ○ | ○ |
| Look for solutions (16) | ○ | ○ | ○ | ○ | ○ |
| Try to accept the situation (17) | ○ | ○ | ○ | ○ | ○ |
| Think of things I can do about it (18) | ○ | ○ | ○ | ○ | ○ |
| Make sure no one can tell what I am feeling (19) | ○ | ○ | ○ | ○ | ○ |
| Pretend that there is no problem (20) | ○ | ○ | ○ | ○ | ○ |

| | | | | | |
|---|---|---|---|---|---|
| Try to express my feelings (21) | ○ | ○ | ○ | ○ | ○ |
| Tell myself that it is all my fault (22) | ○ | ○ | ○ | ○ | ○ |
| Try to think about how I can change my situation (23) | ○ | ○ | ○ | ○ | ○ |
| Worry about the way I was feeling (24) | ○ | ○ | ○ | ○ | ○ |
| Hide my emotions (25) | ○ | ○ | ○ | ○ | ○ |
| Sit with my emotions and let them be (26) | ○ | ○ | ○ | ○ | ○ |
| Think about other ways of seeing the situation I am in (27) | ○ | ○ | ○ | ○ | ○ |
| Focus on slowing my heart rate and breathing (28) | ○ | ○ | ○ | ○ | ○ |
| Distract myself with another task e.g. exercise, TV, or music (29) | ○ | ○ | ○ | ○ | ○ |
| Try to fix it (30) | ○ | ○ | ○ | ○ | ○ |
| Tell somebody how I am feeling (31) | ○ | ○ | ○ | ○ | ○ |
| Act as if nothing is going on (32) | ○ | ○ | ○ | ○ | ○ |
| Do something else to think about something new (33) | ○ | ○ | ○ | ○ | ○ |
| Accept my emotions (34) | ○ | ○ | ○ | ○ | ○ |
| Close myself off to others (35) | ○ | ○ | ○ | ○ | ○ |
| Think about a happier time in my life (36) | ○ | ○ | ○ | ○ | ○ |
| Take some deep breaths (37) | ○ | ○ | ○ | ○ | ○ |
| Worry about what I might do (38) | ○ | ○ | ○ | ○ | ○ |
| Show my feelings through words or actions (39) | ○ | ○ | ○ | ○ | ○ |
| Think about the mistakes I have made (40) | ○ | ○ | ○ | ○ | ○ |
